# Supplementary material for: Stochastic priming and spatial cues orchestrate heterogeneous clonal contribution to mouse pancreas organogenesis
Source: Nat Commun. 2017 Sep 19;8:605. doi: 10.1038/s41467-017-00258-4 (PMC5605525; doi:10.1038/s41467-017-00258-4)
Supplement: Supplementary file 1 — Supplementary information [file 41467_2017_258_MOESM1_ESM.pdf]

File Name: Peer Review File
Description:

File Name: Supplementary Information
Description: Supplementary Figures, Supplementary Tables.

File Name: Supplementary Software
Description: This folder contains all the code necessary to generate and analyze the simulation data for the experiment. The code is written in python3, and is supplied in both jupyter notebook (.ipynb), python (.py) and HyperText Markup Language (.html) format. The readme file provides information on how to use the software.

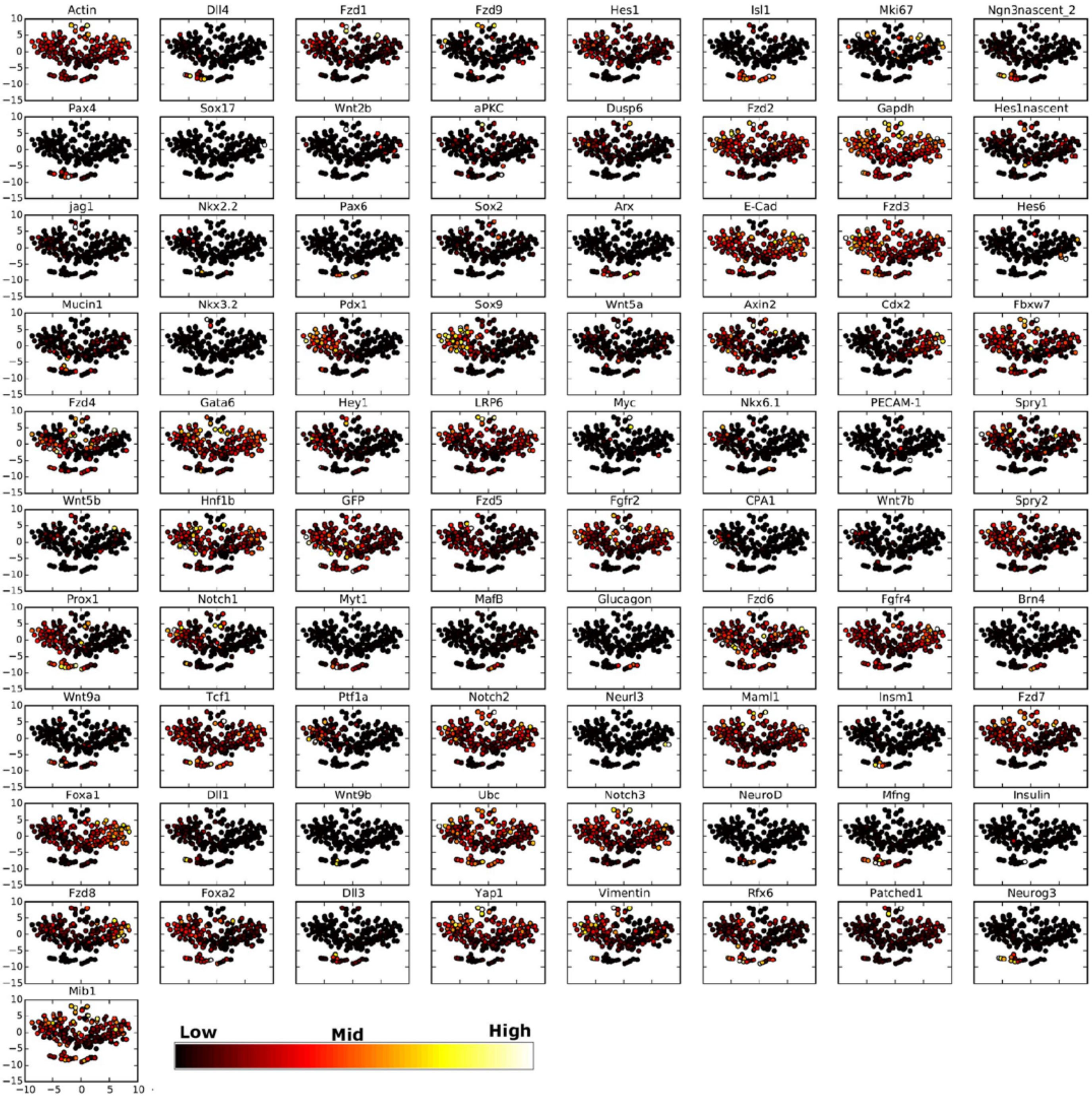

Supplementary Figure 1. Gene expression levels in single cells. tSNE distribution of all single cells and associated normalized expression levels of all functional gene targets.

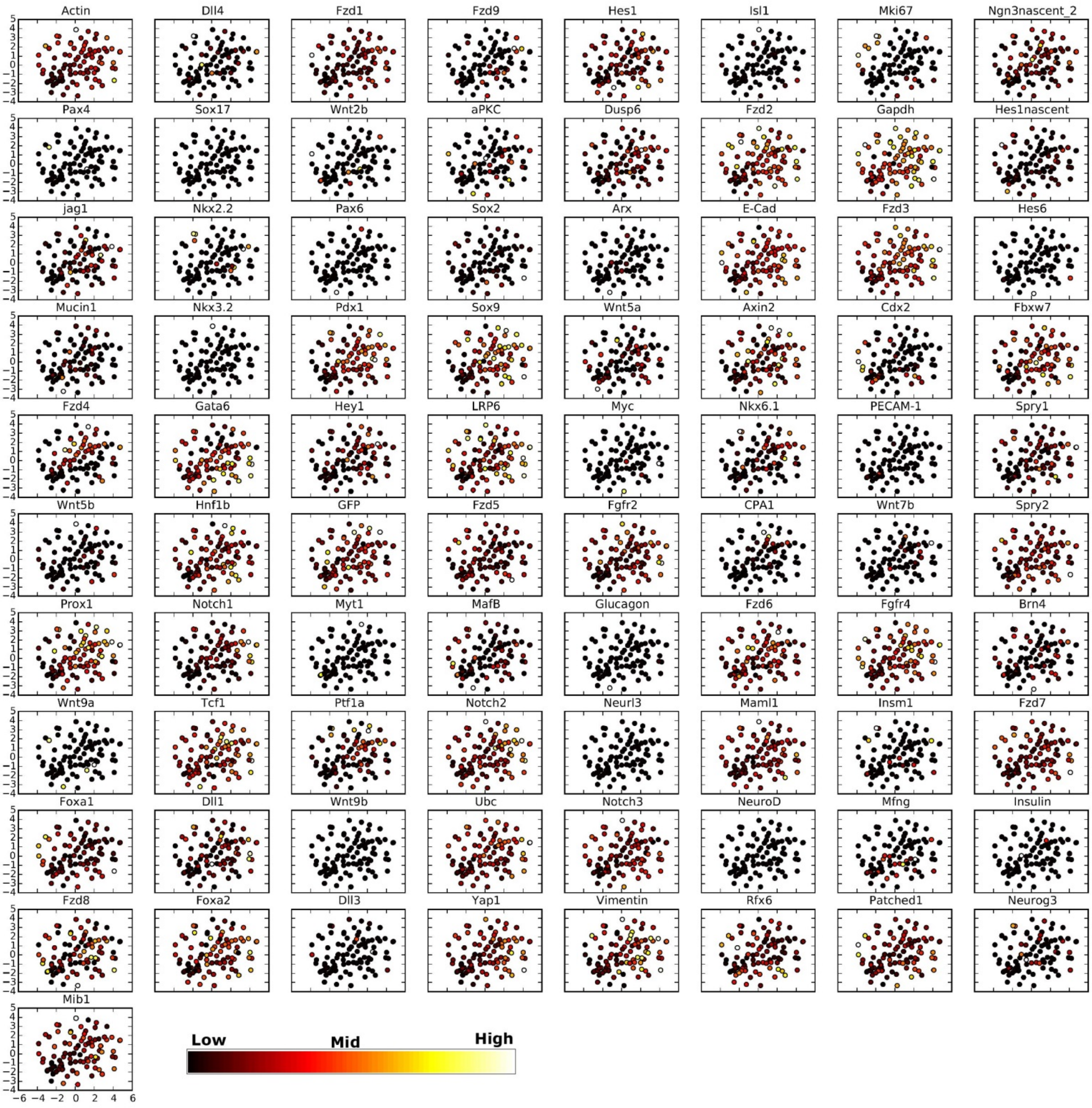

Supplementary Figure 2. Gene expression levels in *Pdx1*⁺ pancreatic progenitors. tSNE distribution of *Pdx1*⁺ progenitors and associated normalized expression levels of all functional gene targets.

Pdx1	Correlation
Notch1	0.607
Sox9	0.548
Nkx6.1	0.481
Prox1	0.477
Fzd3	0.472
Fbxw7	0.449
Ptf1a	0.428
Yap1	0.39
Cdx2	-0.356
Isl1	-0.304
Myt1	-0.261
Wnt9b	-0.203
MafB	-0.189
Pax6	-0.18

Sox9	Correlation
Pdx1	0.548
Gapdh	0.522
Spry1	0.518
Prox1	0.491
E-Cad	0.402
Yap1	0.386
Fzd7	0.345
Notch1	0.336
Isl1	-0.357
Dll3	-0.294
Pax6	-0.288
Myt1	-0.283
Glucagon	-0.272
Wnt9b	-0.271

Hes1	Correlation
Fzd7	0.299
Notch2	0.285
Sox9	0.266
Fzd1	0.266
Spry1	0.253
Yap1	0.218
Myt1	-0.321
Dll3	-0.277
Pecam-1	-0.273
Wnt9b	-0.217
Mfng	-0.216
Pax6	-0.21
Glucagon	-0.209

Hnf1b	Correlation
Notch3	0.422
Foxa1	0.355
Yap1	0.34
E-Cad	0.336
Rfx6	0.301
Nkx3.2	-0.392
Dll3	-0.318

Ptf1a	Correlation
Pdx1	0.428
Fbxw7	0.36
Notch1	0.349
Yap1	0.324
Mfng	-0.305
Neurog3	-0.279
Myt1	-0.255

Nkx6.1	Correlation
Pdx1	0.481
Notch1	0.404
Hey1	0.375
Prox1	0.318
Cdx2	-0.307
Fzd6	-0.236

Neurog3	Correlation
Dll4	0.577
Dll3	0.515
Neurog3 nascent	0.49
Mfng	0.482
Pax4	0.426
Myt1	0.414
Wnt9a	0.261
Wnt9b	0.321
Insm1	0.315
Dll1	0.292
Foxa1	-0.338
Cdx2	-0.31
Dusp6	-0.292
Fzd7	-0.289
Ptf1a	-0.279

Mfng	Correlation
Myt1	0.631
Pax4	0.626
Dll3	0.516
Arx	0.497
Neurog3	0.482
Dll4	0.424
Pax6	0.385
Wnt9a	0.34
Fzd7	-0.574
Yap1	-0.442
Notch2	-0.38
Fzd5	-0.363
Fgfr2	-0.36

Dll1	Correlation
Notch1	0.345
Fgfr2	0.344
Pdx1	0.334
Actin	0.294
Neurog3	0.292
Neurog3 nascent	0.277
Cdx2	-0.312

Supplementary Figure 3. Gene correlations of selected genes. The correlation coefficients of top correlating (blue) and anti-correlating (red) genes with selected pancreatic progenitor and endocrine precursor markers.

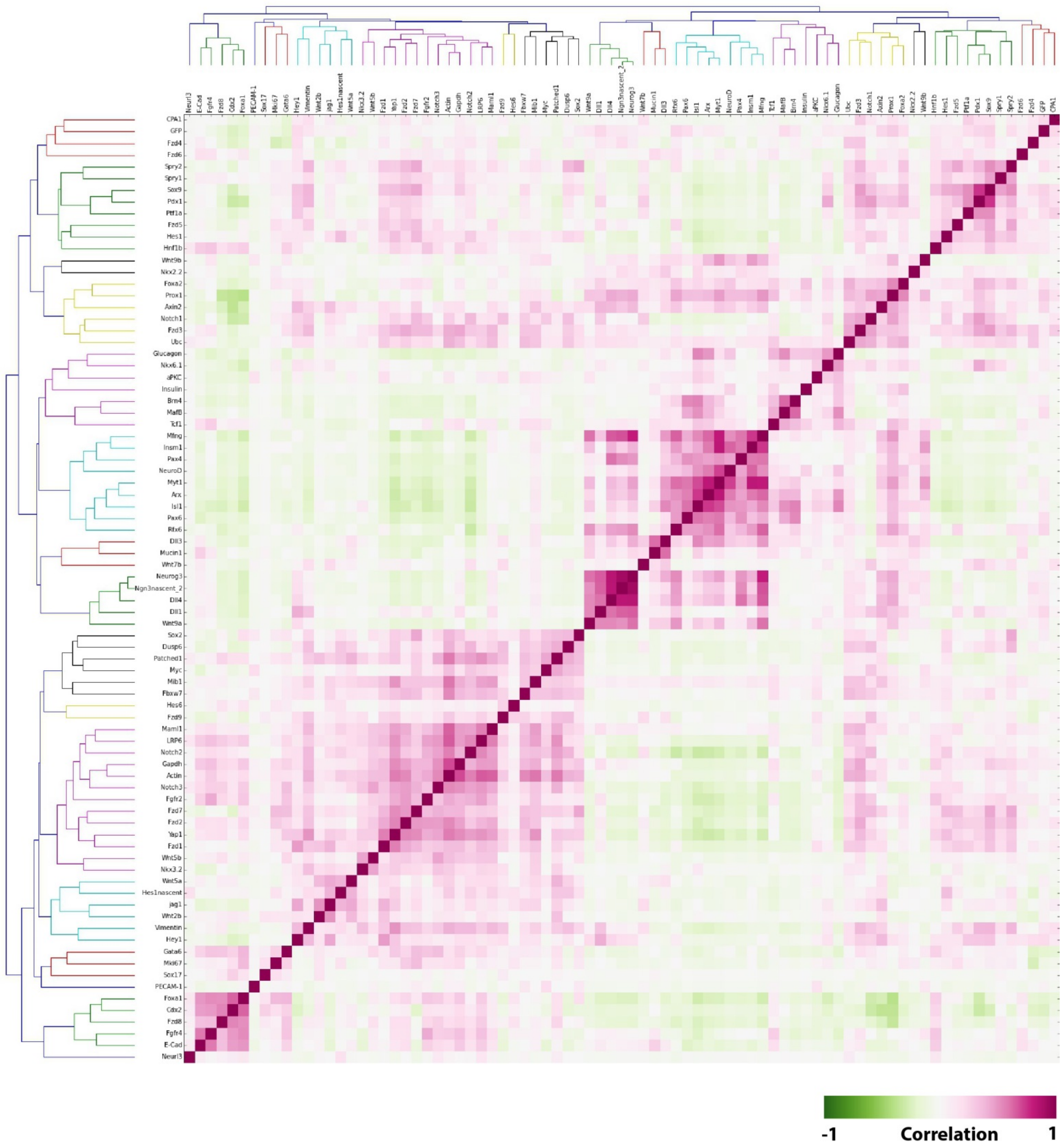

Supplementary Figure 4. Unsupervised hierarchical clustering of gene correlations of all targets. Note the clustering of genes associated with distinct phases of endocrine priming, endocrine differentiation onset, endocrine maturation as well as clusters associated with the pancreatic progenitor state.

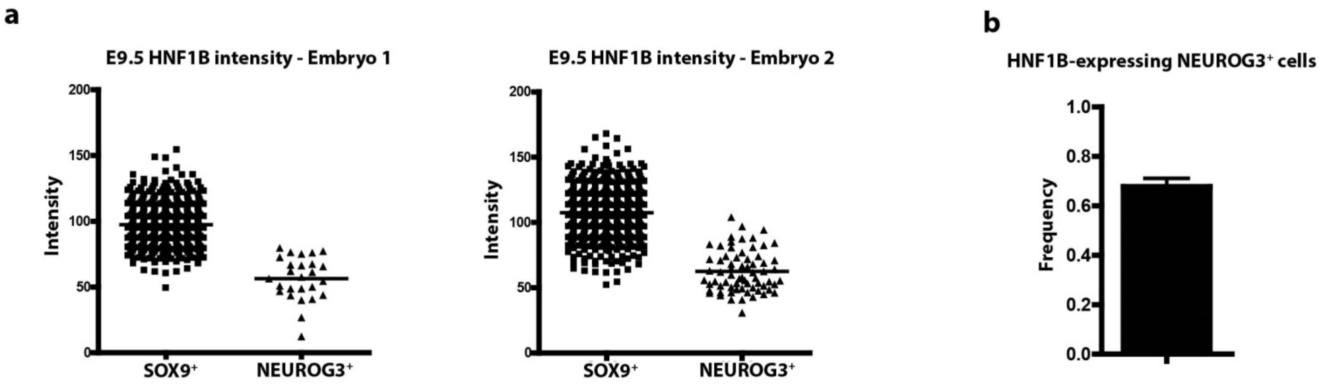

Supplementary Figure 5. HNF1B expression in E9.5 pancreatic progenitors and endocrine precursors. (a) HNF1B immunofluorescence intensity in SOX9^{+} pancreatic progenitors and NEUROG3^{+} endocrine precursors in two separate embryos. (b) The frequency of HNF1B-expression NEUROG3^{+} cells from the two embryos. Mean frequency and standard deviation is indicated. The lowest detectable HNF1B intensity value in SOX9^{+} progenitors was used to set the threshold for scoring NEUROG3^{+} cells as HNF1B^{+} or HNF1B^{-} in individual embryos (n=2).

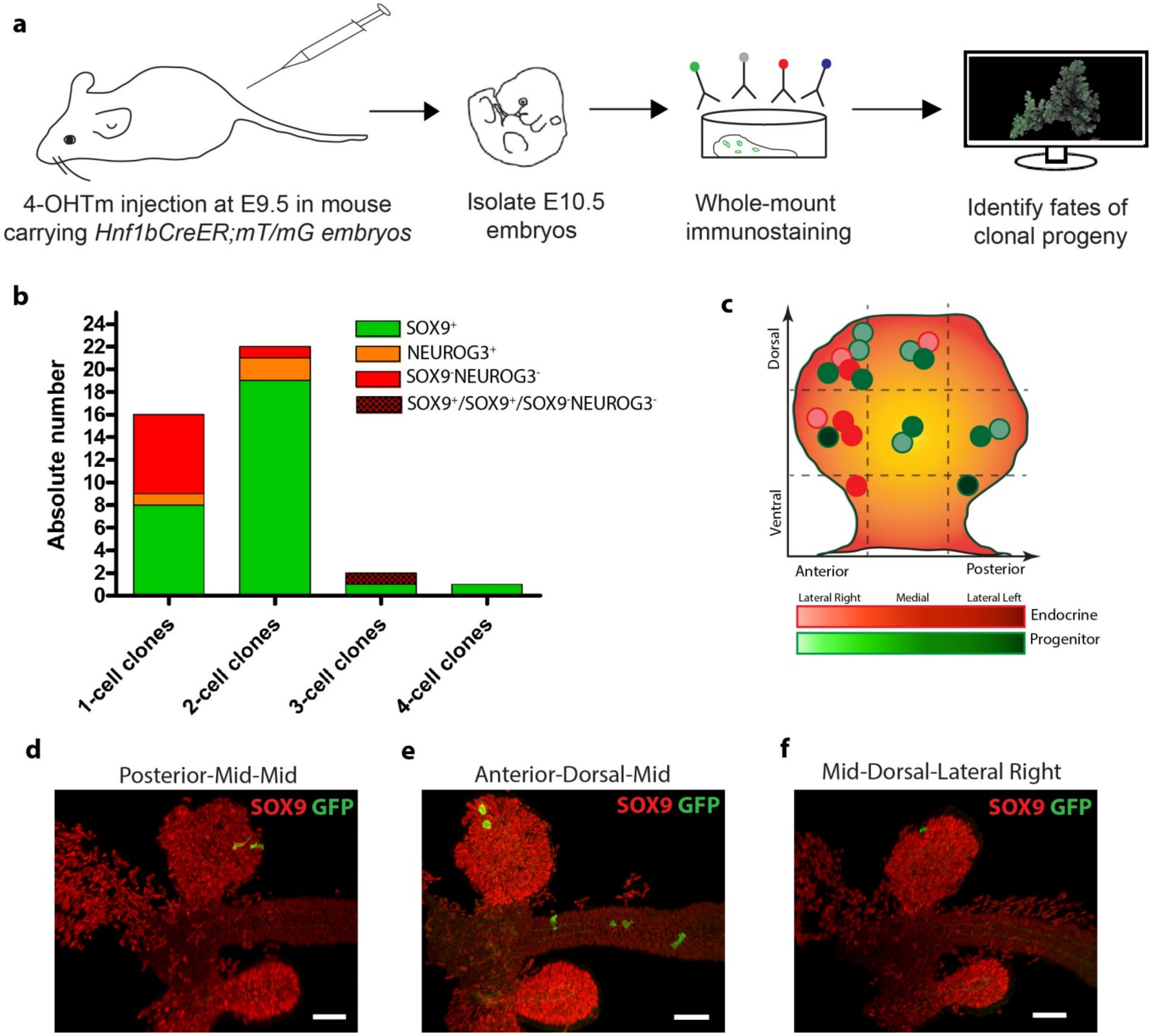

Supplementary Figure 6. E9.5-E10.5 *Hnf1b^{CreER}*-based lineage tracing reveals absence of spatial bias in induction of GFP^{+} progeny. (a) Schematic overview of strategy employed to trace the fate and spatial location of progenitors labelled at E9.5. (b) Quantification of clone size and cell fates at E10.5. GFP^{+} cells were scored as clonal if present within a radius of 30 µm as previously reported in Kim et al. 2015[1]. (c) Spatial map of GFP^{+} location according to cellular fate. The map incorporates data from single-cell and two-cell clones (n=19). Clone position within the E10.5 bud was scored in the dorso-ventral, anterior-posterior and lateral-medial axes and placed into the respective by arbitrary jittering. (d-f) 3D MIP examples of clonal progeny displaying the spatial location indicated above each image. Note that GFP^{+} cells are present in the duodenum in the middle panel as the expression pattern of *Hnf1b* permits *Hnf1b^{CreER}*-mediated lineage tracing in the duodenum. Scale bars, 50 µm.

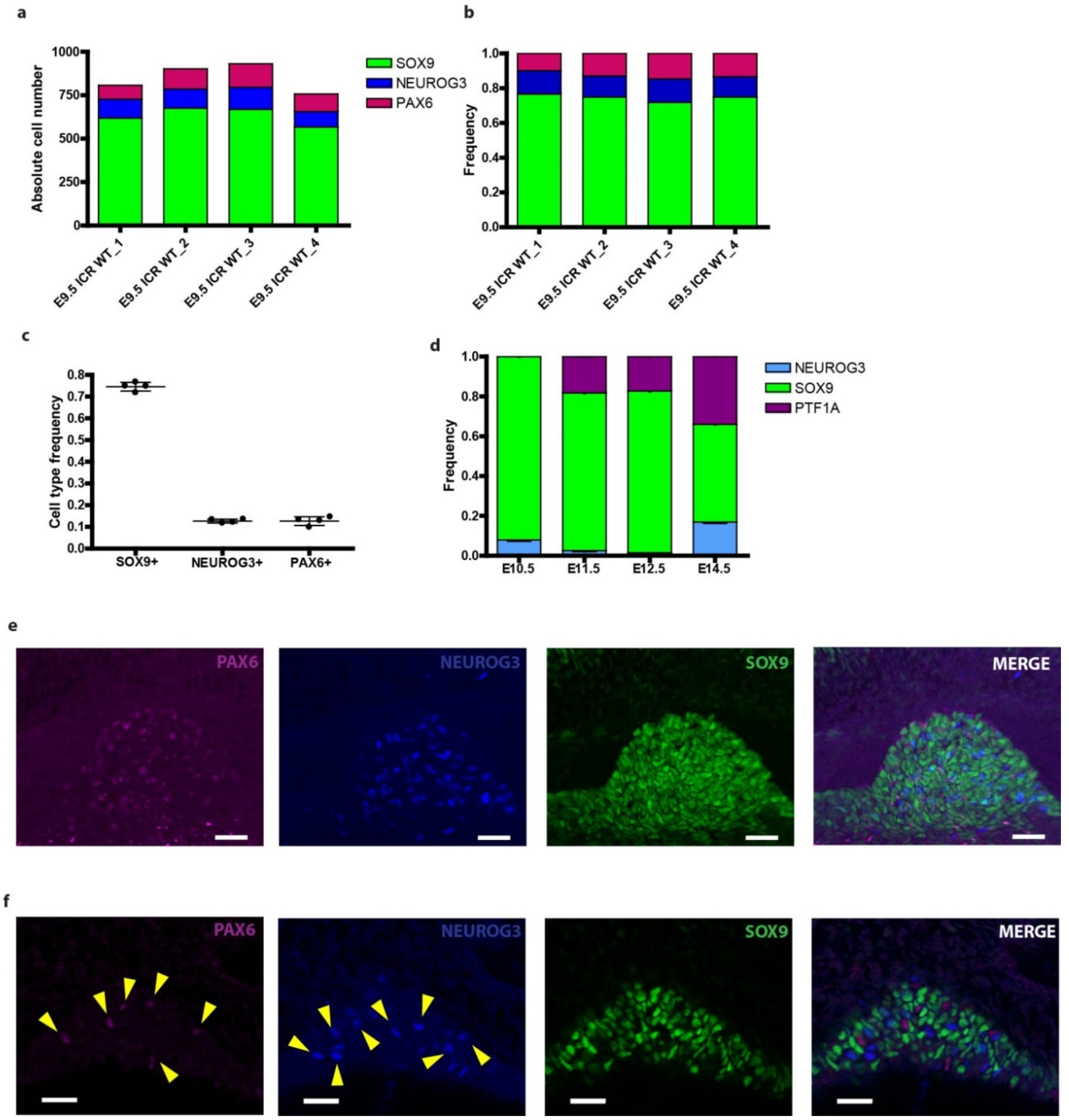

Supplementary Figure 7. Quantification of cell proportions at E9.5. (a) Absolute number of SOX9$^+$ progenitors, NEUROG3$^+$ endocrine precursors and PAX6$^+$ endocrine cells in E9.5 WT dorsal buds in 4 embryos. All cells were manually counted in 3D using Imaris^{TM} software. (b) Frequency of indicated cell types in the same embryos as indicated in (a). (c) Compiled distribution of different cell types from the same samples. Mean frequency and standard deviation are indicated. (d) Frequency of indicated cell types at various stages (n=2 per stage) of pancreas development. For E11.5 and E12.5, PTF1A cells were identified as cells expressing high levels of PTF1A (see materials and methods). Scale bars, 30 µm. (e) 3D MIP example of staining for the indicated markers at E9.5. (f) Optical section from same embryo as in (e) indicating detection of PAX6$^+$ and NEUROG3$^+$ cells (arrowheads). Scale bars, 20 µm.

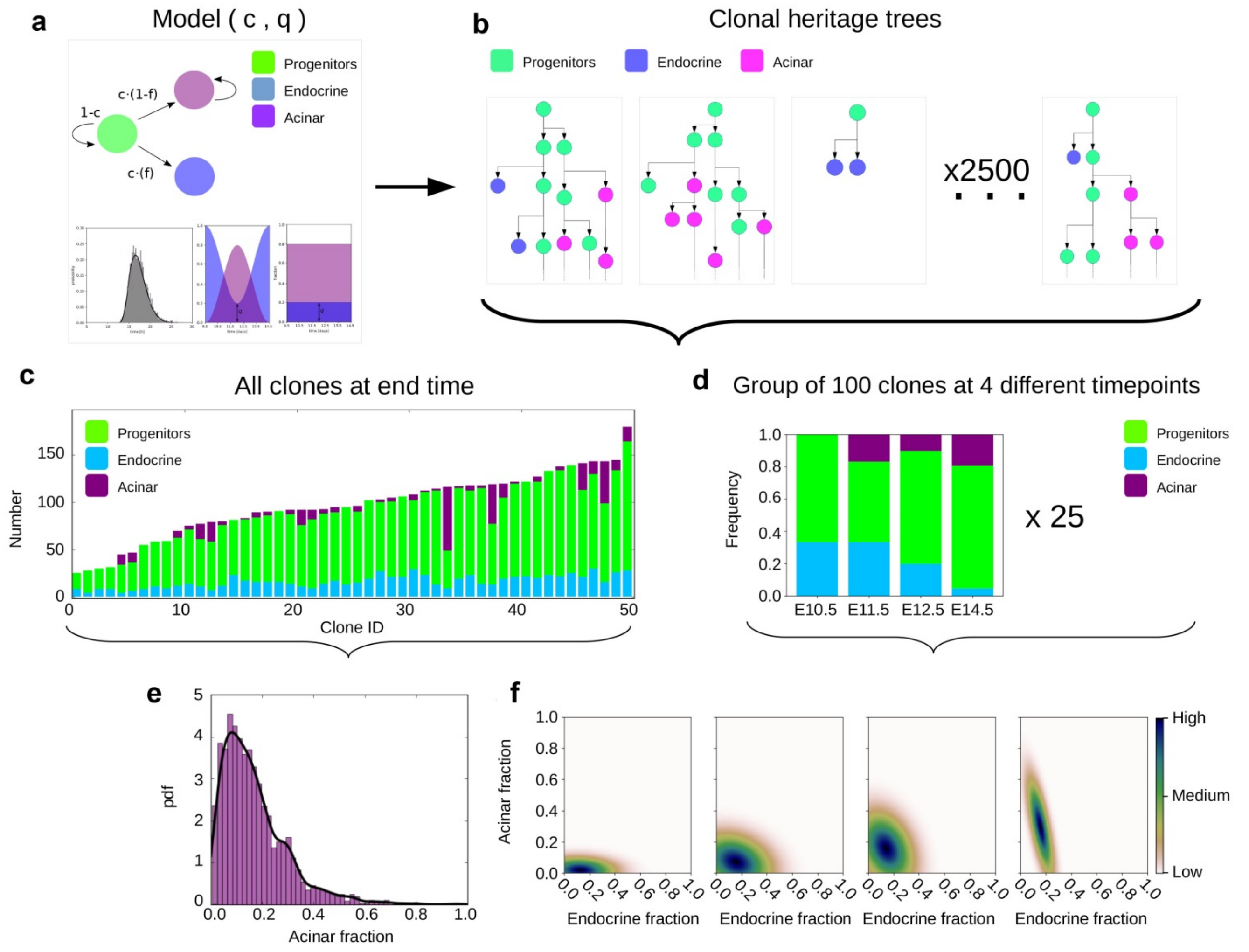

Supplementary figure 8. Overall workflow from model simulations to approximate probability density functions (PDFs). (a) For a single model (either Model 1 or Model 2), a certain cell cycle distribution and one value of parameters c and q we can simulate how one progenitor clone expands for a period of 5 days, starting at E9.5. The color-scheme in (a)-(e) is the same as in Fig. 5: progenitors (green), endocrine(blue) and acinar (purple). (b) For each parameter set (c, q) we simulate 2500 expanding progenitor clones. (c) Based on the lineage for each of these simulated clones we make a histogram of clonal compositions at E14.5. Here only 50 clones are shown for simplicity. (d) From the histogram (c) we select all lineages with at least one purple cell and use a KDE to approximate the PDF underlying simulation results (shown in black). For comparison the PDF has been shown together with a histogram of the purple acinar fractions. (d) For groups each consisting of 100 in silico clones we have calculated the factions of different types of cells at time points E10.5, E11.5, E12.5 and 14.5 (shown it as a bar chart). (f) Using both the acinar and endocrine fractions for each time point for the 25 groups (each of 100 simulated clones) we estimate the PDFs of having certain combinations of acinar and endocrine fraction. The color-scale ranges from black (highest values) to blue, green , brown and white (lowest values).

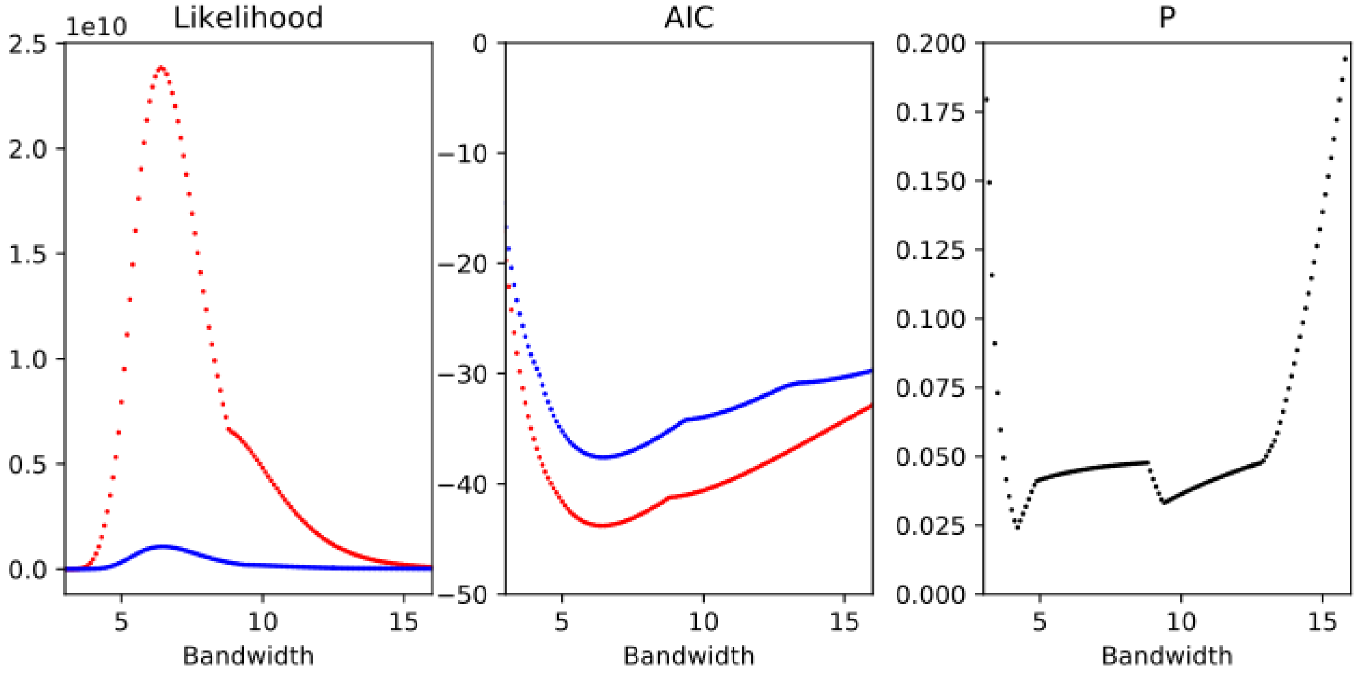

Supplementary figure 9. Kernel Density Estimations of PDFs. Left: Likelihood, L, that Model 1 (blue) and Model 2 (red) fit the data for their respective best parameter sets as a function of KDE bandwidth. Both models have a clear maximum at bandwidth of 6.5. Middle: Akaike Information Criterion (AIC) score for both models as a function of bandwidth. In line with likelihood, the minima in AIC is at bandwidth of 6.5. Right: Probability, P, that Model 1 and Model 2 are equally good.

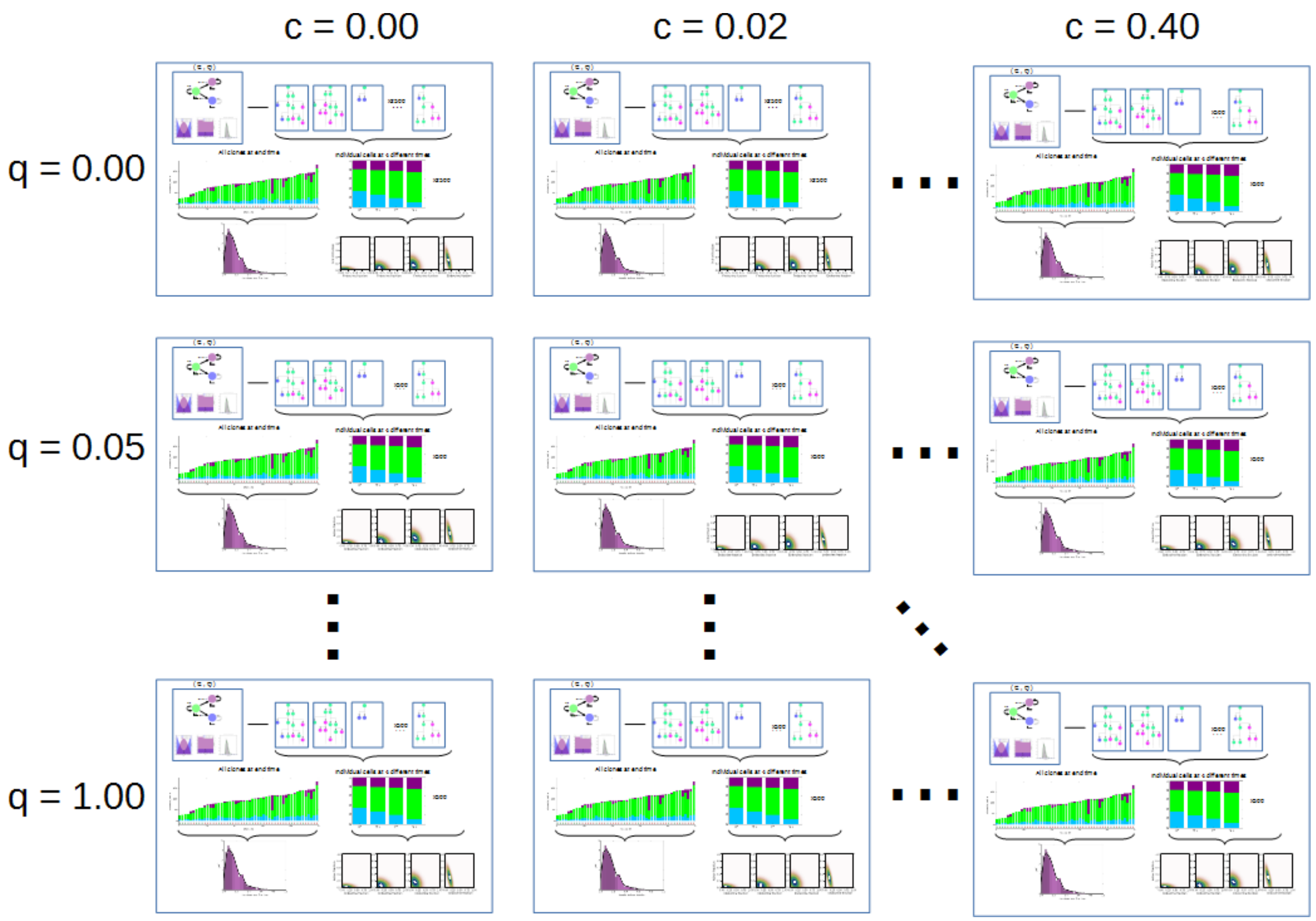

Supplementary figure 10. Scanning parameter space for differentiation and endocrine versus acinar fate. The parameter scan ranges from 0 to 0.4 in c and 0 to 1 in q, with 20 intervals. For each parameter combination we generate a model 1 and model 2 and generate 2500 progenitor cells at time E9.5. From here we do the same procedure as shown in Supplementary Fig. 8.

Antigen	Host species	Supplier	Dilution
CPA1	Goat	R & D systems	1/500
E-CADHERIN	Mouse	Sigma Aldrich	1/200
GFP	Chick	AbCam	1/1000
HES1	Rabbit	Cell Signaling	1/1000
HNF1B	Goat	Santa Cruz	1/500
NEUROG3	Goat	BCBC	1/1000
PAX6	Mouse	DSHB	1/400
PDX1	Goat	BCBC	1/500
PTF1A	Rabbit	BCBC	1/2000
SOX9	Rabbit	Millipore	1/2000

BCBC: Beta Cell Biology Consortium
DHSB: Developmental Studies Hybridoma Bank

Supplementary table 1: List of antibodies

Target	Forward sequence	Reverse sequence
Actin	CCCTAAGGCCAACCGTGAAA	CAGCCTGGATGGCTACGTAC
aPKC	GCATCCAGACCACACACAGA	TTGCCTCCTTTTCTTCACCA
Arx	TTTCCAGAAGACGCACTACCC	TCTGTCAGGTCCAGCCTCA
Axin2	GATCCACGGAAACAGCTGAA	AGCCGGAACCTACGTGATAA
Brn4	AGGCCTTACAACTGAGCTTCA	GGATGAATCAGCCTCCTCCA
Cdx2	CGATACATCACCATCAGGAGGAA	TGGCTCTGCGGTTCTGAAA
Cpa1	GCAAAACTCGATCACACACC	CTACTAGCTCCGGGCATCC
Dll1	CACCAAGTACCAGTCGGTGT	TCCATCTTACACCTCAGTCGC
Dll3	TGCACGCCATTCCCAGAC	CGGCATTCATCAGGCTCTTCA
Dll4	GAGAAGGTGCCACTTCGGTTA	TAGAGTCCCTGGGAGAGCAAA
Dusp6	GCTGCTGCTCAAGAAACTCA	TCGGCCTGGAACTTACTGAA
E-Cadherin	ATTGCAAGTTCCTGCCATCC	CAGTAGGAGCAGCAGGATCA
Fbxw7	AAAAGTTTGGTCAGCGGTCAC	TCATCTGTGATGACCACACTCC
Fgfr2	TCAAGTGGATGGCTCCTGAA	CACATTAACACCCCGAAGGAC
Fgfr4	CTTCCACGGGGAGAATCGTA	CGAGGGTACCACACTTTCCA
Foxa1	TGAGAGCAACGACTGGAACA	CCGGAGTTCATGTTGCTGAC
Foxa2	TGAAGATGGAAGGGCACGAG	CACGGAAGAGTAGCCCTCGG
Fzd1	CACGGTGCTCACGTACCTA	TGTAACAGCCGGACAGGAAA
Fzd2	ACTCTGGAGCACCCTTTCCA	CGCGCTCACCCAGAAACTTATA
Fzd3	TGACCAACAGACTGCAGCTTTA	ATGGCCGAAAATCCCGAGAA
Fzd4	CTTTCACGCCGCTCATCC	TGGCACATAAACCGAACAAA
Fzd5	GTTGCCACCTTCCTCATTGAC	AGGTAGCACGCAGACAAGAA
Fzd6	CGATGGCCTGAAGAACTTGAA	AGCTCTGTGTGTGGATGAGAA
Fzd7	GAGGTGCACCAGTTCTACCC	CGGGTGCGTACATAGAGCATAA
Fzd8	TCTACAACCGCGTCAAGAC	CGCTCATCCTGGCTAAAGAA
Fzd9	CGGTTTTGTGGCTCTCTTCC	TCAGCTTCTCCAGCTTCTCC
Gapdh	AGACGGCCGCATCTTCTT	TTCACACCGACCTTCACCAT
Gata6	CCCCTCATCAAGCCACAGAA	AGGTAGTGGTTGTGGTGTGAC
GFP	TTCAAGGACGACGGCAACTA	TCAGCTCGATGCGGTTCA
Glucagon	TGTGCAGTGGTTGATGAACA	CCTTCAGCATGCCTCTCAAA
Glut2	CTCCAGGAAGGGTGCTAAACC	TGCTCCCTATCCGTTCTTCAA
Hes1	ACTTAAGAAAGATAGCTCCCGGC	TCCGGAGGTGCTTCACAGTC
Hes1 nascent	ACACCGGACAAACCAAAGAC	GCTGTGCACAACCCACTTAA
Hes6	GAGGATGAGGACCGCTGG	AGACTCTCGTTGATCCGTGC
Hey1	ACGAGACCATCGAGGTGGAA	CGTTGGGGACATGGAACACA
Hnf1b	CGGCAAAAGAATCCCAGCAA	AGACCCCTCGTTGCAAACA
Ins2	ACCCACAAGTGGCACAACTG	TACAATGCCACGCTTCTGCT
Insm1	GTGTTCCCCTGCAAGTACTG	TCTATTCTCAGACGGGTGGC
Isl1	GGACAAGAAACGCAGCATCA	GTTCCTGTCATCCCCTGGATA
Jag1	CTTCAATCTCAAGGCCAGCC	CACCAGCAAAGTGTAGGACC
Lrp6	CCCCATGCACTTAGTTCTGC	CTGCTGAGGAGAACACGTTG
MafB	GGCAACTAACGCTGCAACTCT	CAACGGAAGGGACTTGAACAC
Maml1	CACAAAGCACCTTCCCACAA	CGGACCCAAGTTGTTCATCC
Mfng	TTTCTGCATCAACCGCCAAC	TGAGAGCAGAAGTGTCCACAAA

Supplementary table 2: Primers used for single cell qPCR

Target	Forward sequence	Reverse sequence
Mib1	GTTCTCTGGGATAACGGTGCTA	CTTTGGCATCCTGGACACAC
Mki67	GAGACATACCTGAGCCCATCA	GCTTTGCTGCATTCCGAGTA
Mucin1	AGTACCAAGCGTAGCCCCTA	CACCACAGCTGGGTTGGTATAA
Myc	AGTGCTGCATGAGGAGACA	TCTCCACAGACACCACATCAA
Myt1	CTTGCCAATATTCGCCTTCCA	TGTCAGTGAGGGTGTTCACA
Neurl3	TTCGTAGTGGTGAGCCTGTG	GTGTTGGCAGTGTTGTGGAA
NeuroD	GCTCCAGGGTTATGAGATCGT	GCTCTCGCTGTATGATTTGGT
Neurog3	GACTGACCTGCTGCTCTCTA	GCTCCGGGAAAAGGTTGTTG
Ngn3 nascent	GACTGACCTGCTGCTCTCTA	CATCCACCCTTTGGAGTTTCC
Nkx2.2	ACCGAGGGCCTCCAATACTC	CCTTGTCATTGTCCGGTGACT
Nkx3.2	AGCGACAGCGAGATGTCA	TGCACCTCCAGGGCTAAC
Nkx6.1	GGCCTATTCTCTGGGGATGAC	GCTGCGTGCTTCTTTCTCC
Notch1	AGATGCTCAGGGTGTCTTCC	GTGGAGTTGTGCCATCATGC
Notch2	TGGTTCTGGGACAAGTGAACA	ACAGCAAAGCCTCATCCTCA
Notch3	CCATGCCGATGTCAATGCA	TAGCCTCCACGTTGTTCACA
Patched1	TGCTGGAGGAGAACAAGCAA	CCAGTCACTGTCAAATGCATCC
Pax4	ATCCCAGGCCTATCTCCAAC	GGCCAGGCAAATTCCACATA
Pax6	TATCCCGGGACTTCAGTACCA	TGATGGAGTTGGTGTTCTCTCC
Pdx1	TCCCTTTCCCGTGGATGAAA	TCGGGTTCCGCTGTGTAA
Pecam1	GCACAGTGATGCTGAACAAC	GTCACCTTGGGCTTGGATAC
Prox1	GCCCTCAACATGCACTACAAC	CGTGATCTGCGCAACTTCC
Ptf1a	AGGACAGTCCCGGTAACCA	TCAGTCCAGGAAAGAGAGTGC
Rfx6	GGCATCAAAGAAAGCAGTGCATA	TCTTCAGTTTGCTCCCCGAAA
Sox17	GCACAGCAGAACCCAGATCT	GGTCAACGCCTTCCAAGAC
Sox2	TGAAGGAGCACCCGGATTATA	CGGGAAGCGTGTACTTATCC
Sox9	AGTACCCGCATCTGCACAA	GTCTCTTCTCGCTCTCGTTCA
Spry1	ACTGCACCAAGACCCGAAAA	GGTGCTCGTAGCTGCTATTCA
Spry2	GGAGAGGGGTTGGTGCAAA	AGGTCTTGGCAGTGTGTTCA
Tcf1	AGGCCGAGATGCTATCTTCA	TGCAGGGCTACTCTTGTCA
Ubc	GTCTGCTGTGTGAGGACTGC	CCTCCAGGGTGATGGTCTTA
Vimentin	GATTTCTCTGCCTCTGCCAAC	CAACCAGAGGAAGTGACTCCA
Wnt2b	CTTTGACAACTCCCCTGACTAC	TTCACACCCATCTGTTCCTTT
Wnt5a	ACACAACAATGAAGCAGGCC	GAGCCAGACACTCCATGACA
Wnt5b	ACCTACAGAACAACGAGGCT	GACTCCGTGACATTTGCAGG
Wnt7b	CGCCTCATGAACCTTCACAAC	CCTGACACACCGTGACACTTA
Wnt9a	CGAGTGGACTTCCACAACAA	GGCATTTGCAAGTGGTTTCC
Wnt9b	GGCCCAAGAGAGGAAGCA	GCACTTGCAGGTTGTTCTCA
Yap1	CTGCCCGACTCCTTCTTCAA	CCGCAGTACCTGCATCAGTA

Supplementary table 2 continued: Primers used for single cell qPCR

Supplementary reference

1. Kim YH, Larsen HL, Rue P, Lemaire LA, Ferrer J, Grapin-Botton A. Cell cycle-dependent differentiation dynamics balances growth and endocrine differentiation in the pancreas. PLoS biology 13, e1002111 (2015).
