# Supplementary material for: Stochastic priming and spatial cues orchestrate heterogeneous clonal contribution to mouse pancreas organogenesis
Source: Nat Commun. 2017 Sep 19;8:605. doi: 10.1038/s41467-017-00258-4 (PMC5605525; doi:10.1038/s41467-017-00258-4)
Supplement: Supplementary file 2 — Supplementary Software [file 41467_2017_258_MOESM2_ESM.zip › code/simulation_5.html]

simulation\_5


# Import statements¶

In [2]:

```
# init
import os, sys
sys.path.insert(0,os.path.pardir)
import pandas as pd

import collections
import matplotlib.pylab as plt
from matplotlib import interactive
interactive(False)

import scipy.signal

#import mpld3
#mpld3.enable_notebook()

import numpy as np
from pprint import pprint
import scipy
import scipy.special
import scipy.stats
from random import random
from collections import defaultdict
from copy import copy, deepcopy
import colorama
from colorama import Fore, Back, Style

from scipy.stats import norm
from sklearn.neighbors import KernelDensity

from tqdm import tqdm, tqdm_notebook, tnrange
from statsmodels.distributions.empirical_distribution import ECDF

#%load_ext line_profiler
#%matplotlib notebook

from scipy.interpolate import interp1d
from scipy.stats import norm, gamma

figwidth = 10
figheight = 5
```

# Misc Func definitions¶

In [3]:

```
# Visualize f(t) models given two plotting areas ax1 and ax2
def plot_f_of_t(blue_probab_sine, purple_probab_sine,blue_probab_line, purple_probab_line, blue_probab_flat, purple_probab_flat, ax1, ax2, ax3, q = 0.2):
    q = q
    start_height_blue = 1; min_height_blue = q
    shb = start_height_blue; mhb = min_height_blue
    arrowheight = 0.03
    t = np.linspace(0,1,101)
    
    ax1.set_title('model 1')
    ax1.clear()
    ax1.fill_between(t, blue_probab_sine(t,mhb) , alpha = 0.5, color = 'blue'  , label = 'G -> B, ($f$ )')
    ax1.fill_between(t, purple_probab_sine(t,mhb), alpha = 0.5, color = 'purple', label = 'G -> P, ($1-f$ )')
    ax1.set_xlabel('time'); ax1.set_ylabel('fraction'); ax1.legend()

    ax1.arrow(0.5, 0,   0, mhb -arrowheight, head_width=0.03, head_length=arrowheight, fc='k', ec='k')
    ax1.arrow(0.5, mhb, 0, -mhb+arrowheight, head_width=0.03, head_length=arrowheight, fc='k', ec='k')
    ax1.text(0.52, mhb/2-0.02, r'$q$', fontsize=15)

    ax2.set_title('model 2'); 
    ax2.clear()
    ax2.fill_between(t,  blue_probab_line(t,mhb) , alpha = 0.5, color = 'blue'  , label = 'G -> B, ($f$ )')
    ax2.fill_between(t,purple_probab_line(t,mhb), alpha = 0.5, color = 'purple', label = 'G -> P, ($1-f$ )')
    ax2.set_xlabel('time'); ax2.set_ylabel('fraction'); ax2.legend()

    ax2.arrow(0.5, 0,   0, mhb -arrowheight, head_width=0.03, head_length=arrowheight, fc='k', ec='k')
    ax2.arrow(0.5, mhb, 0, -mhb+arrowheight, head_width=0.03, head_length=arrowheight, fc='k', ec='k')
    ax2.text(0.52, mhb/2-0.02, r'$q$', fontsize=15)
                     
    ax3.set_title('model 2'); 
    ax3.clear()
    ax3.fill_between(t,  blue_probab_flat(t,mhb) , alpha = 0.5, color = 'blue'  , label = 'G -> B, ($f$ )')
    ax3.fill_between(t,purple_probab_flat(t,mhb), alpha = 0.5, color = 'purple', label = 'G -> P, ($1-f$ )')
    ax3.set_xlabel('time'); ax3.set_ylabel('fraction'); ax3.legend()

    ax3.arrow(0.5, 0,   0, mhb -arrowheight, head_width=0.03, head_length=arrowheight, fc='k', ec='k')
    ax3.arrow(0.5, mhb, 0, -mhb+arrowheight, head_width=0.03, head_length=arrowheight, fc='k', ec='k')
    ax3.text(0.52, mhb/2-0.02, r'$q$', fontsize=15)
    return ax1, ax2

# Plotting the division distributions given a drawing area ax3
def plot_division_distributions(cell_cycle_dist_A, cell_cycle_dist_loc_A, cell_cycle_dist_scale_A, cell_cycle_dist_P, cell_cycle_dist_loc_P, cell_cycle_dist_scale_P, ax3):
    ax3.set_title('Distribution');
    ax3.clear()
    t_n = np.linspace(0,100,1000)

    # Plot the actual distribution
    if 1 == 1:
        ax3.plot(t_n, scipy.stats.gamma.pdf(t_n,
                                            a     = cell_cycle_dist_A,
                                            loc   = cell_cycle_dist_loc_A,
                                            scale = cell_cycle_dist_scale_A), 
                 linewidth = 2, color = 'purple', label = 'purple')
        ax3.plot(t_n, scipy.stats.gamma.pdf(t_n,
                                            a     = cell_cycle_dist_P,
                                            loc   = cell_cycle_dist_loc_P,
                                            scale = cell_cycle_dist_scale_P), 
                 linewidth = 2, color = 'green' , label = 'green')
        ax3.plot(t_n, t_n*0, linewidth = 2, color = 'blue'  , label = 'blue')

    # Define rand-generator
    def green_dist(size = 1):
        return scipy.stats.gamma.rvs(a     = cell_cycle_dist_P,
                                     loc   = cell_cycle_dist_loc_P,
                                     scale = cell_cycle_dist_scale_P,
                                     size = size)
    def purple_dist(size = 1):
        return scipy.stats.gamma.rvs(a     = cell_cycle_dist_A,
                                     loc   = cell_cycle_dist_loc_A,
                                     scale = cell_cycle_dist_scale_A,
                                     size = size)

    # Make histogram of rand-generated nums - Green
    r_g = green_dist(size  = 1000);
    ax3.hist(r_g,bins = 50, normed=True, histtype='stepfilled', alpha=0.2, color = 'green');

    # Make histogram of rand-generated nums - Purple
    r_p = purple_dist(size=1000);
    ax3.hist(r_p,bins = 50, normed=True, histtype='stepfilled', alpha=0.2, color = 'purple');

    #plt.legend()
    ax3.set_title('Division distibution')
    ax3.set_xlabel('time [h]')
    ax3.set_ylabel('probability')
    ax3.set_xlim(5.0, 35.0);
    ax3.set_ylim(0.0,  0.3);
    return ax3

# Sorts all the results series of on parameter set and groups the clones of each simulation into 3 types
def sort_and_group(cell_count_total, cell_count_B, cell_count_G, cell_count_P):
    # Sort according to total
    idx=np.argsort(cell_count_total)
    # Make lists according to sort
    b_for_all = np.array(cell_count_B)[idx]
    g_for_all = np.array(cell_count_G)[idx]
    p_for_all = np.array(cell_count_P)[idx]
    totals = np.array(cell_count_total)[idx]
    # Logics for seperating into the groups.
    group3_bool = p_for_all != 0  # Find all the samples with acinar cells (These will have 3 types of cells)
    group1_bool = b_for_all == totals # Find all the samples that is only endocrine (Only one type of cell)
    group2_bool = (group3_bool | group1_bool) != 1 # Everything else. (This should be Progenitors and Endocrine) (2 types of cells)
    # Indicies for members of each of the groups
    idx_1 = np.array([num for num,elem in enumerate(group1_bool) if elem == True])
    idx_2 = np.array([num for num,elem in enumerate(group2_bool) if elem == True])
    idx_3 = np.array([num for num,elem in enumerate(group3_bool) if elem == True])
    return b_for_all, g_for_all, p_for_all, totals, idx_1, idx_2, idx_3

# Plots the above
def plot_sort_and_group(b_for_all, g_for_all, p_for_all, totals, idx_1, idx_2, idx_3, subplotlayout = 111):
    num_clones = len(totals)
    r_c = np.arange(num_clones) # Range_Clones
    fig = plt.figure(figsize=(12,5))
    ax1 = fig.add_subplot(subplotlayout)

    # Plot the bars
    ax1.bar(r_c,b_for_all,                           color='#00BFFF',lw=0, label = 'B')
    ax1.bar(r_c,g_for_all,bottom=b_for_all,          color='#00FF00',lw=0, label = 'G')
    ax1.bar(r_c,p_for_all,bottom=b_for_all+g_for_all,color='#800080',lw=0, label = 'P')

    ax1.set_xlabel('Clone ID')
    ax1.set_ylabel('Clone size, cell #')

#     # Plot the boxes between the bars
#     ax1.plot(idx_1+0.4,len(idx_1)*[totals.max() * -0.05],'bs')
#     ax1.plot(idx_2+0.4,len(idx_2)*[totals.max() * -0.05],'gs')
#     ax1.plot(idx_3+0.4,len(idx_3)*[totals.max() * -0.05],'rs');
#     ax1.set_ylim([totals.max() * -0.1 , totals.max() * 1.1]);
    return fig, ax1

# Makes a dictionsary that incodes the information about decendants numberand more. Recursive function!
def get_decendants_numbers(cells):
    n_decendants_dict = defaultdict(lambda: 1.0)
    def get_n_offspring(cell_n,cells,n_decendants_dict):
        list_of_offsprings = cells.loc[cell_n,'offspring']
        for number in list_of_offsprings:
            #n_decendants_dict[cell_n] = n_decendants_dict[cell_n] + 1.0
            n_decendants_dict[cell_n] = n_decendants_dict[cell_n] + get_n_offspring(number,cells,n_decendants_dict)
        if len(list_of_offsprings) == 0:
            return 1
        return n_decendants_dict[cell_n]
    get_n_offspring(0,cells,n_decendants_dict)
    return n_decendants_dict

def bootstrap_resample(X, n=None):
    """ Bootstrap resample an array_like 
    Parameters
    ----------
    X : array_like
      data to resample
    n : int, optional
      length of resampled array, equal to len(X) if n==None
    Results
    -------
    returns X_resamples
    """
    if n == None:
        n = len(X)
    resample_i = np.random.randint(0, high=len(X), size=n)
    X_resample = X[resample_i]
    return X_resample
```

# Define and declare the system¶

## Intro text¶

The system can mostely be defined by a transition matrix. A transition matrix of two variables. $c$ and $f$

$$
\begin{bmatrix}
B \rightarrow B & B \rightarrow G & B \rightarrow P \\
G \rightarrow B & G \rightarrow G & G \rightarrow P \\
G \rightarrow B & G \rightarrow G & G \rightarrow P \\
\end{bmatrix}
=
\begin{bmatrix}
1 & 0 & 0 \\
c \cdot f & c & c\cdot(1-f) \\
0 & 0 & 1 \\
\end{bmatrix}
$$

Below is a diagram of the model. The distributions below each state represents cell divisions

The parameter controling the faction of green that goes to blue and purple, $f$, changes over time, while $c$ remains constant.

The change of $f$ is modeled according to the one of the two models below, where a new parameter is introduced. This parameter we call $q$.

So all in all there is 3 parameters; $f$,$c$ and $q$. It should be added here that $f$ is time dependand, $f(t)$, as per the model in use and the parameter $q$

In [4]:

```
fig_model = plt.figure('Model',figsize=(figwidth*1.5,figheight)); 
ax1 = fig_model.add_subplot(1,4,1); ax2 = fig_model.add_subplot(1,4,2); ax3 = fig_model.add_subplot(1,4,3) ; ax4 = fig_model.add_subplot(1,4,4)
fig_model.show()
```

```
/usr/lib/python3.6/site-packages/matplotlib/figure.py:402: UserWarning: matplotlib is currently using a non-GUI backend, so cannot show the figure
  "matplotlib is currently using a non-GUI backend, "
```

## f(t)¶

In [5]:

```
#f(t)
# # # Sinus form # # #
# blue_probab_sine =   lambda t,q: q+(1-q)*(1 - np.sin(t*np.pi)) 
blue_probab_sine =   lambda t,q: q+(1-q)*0.5*(1 + np.cos(t*2*np.pi))
purple_probab_sine = lambda t,q: 1-blue_probab_sine(t,q) # I do not really use these, since they are just 1-blue_probab
# # # Line form # # # 
blue_probab_line =   lambda t,q: q+(1-q)*(2*np.abs(0.5-t)) 
purple_probab_line = lambda t,q: 1-blue_probab_line(t,q) # I do not really use these, since they are just 1-blue_probab
# # # Flat form # # #
blue_probab_flat =   lambda t,q: q 
purple_probab_flat = lambda t,q: 1-blue_probab_flat(t,q) # I do not really use these, since they are just 1-blue_probab

# Visualize f(t) models given two plotting areas ax1 and ax2
ax1, ax2 = plot_f_of_t(blue_probab_sine, purple_probab_sine,blue_probab_line, purple_probab_line,  blue_probab_flat, purple_probab_flat, ax1, ax2, ax3, q = 0.2)
fig_model.show()
```

```
/usr/lib/python3.6/site-packages/matplotlib/figure.py:402: UserWarning: matplotlib is currently using a non-GUI backend, so cannot show the figure
  "matplotlib is currently using a non-GUI backend, "
```

## Division distribution¶

In [6]:

```
# Defining cell cycle lengths

# Distribution of Cell Cycle Lengths A (Purple)
mean_cell_cycle_A = 15 # hours
cell_cycle_dist_A =  7    
cell_cycle_dist_loc_A = 0.8 * mean_cell_cycle_A
cell_cycle_dist_scale_A = 0.05 * mean_cell_cycle_A


# Distribution of Cell Cycle Lengths P (Green)
mean_cell_cycle_P = 15
cell_cycle_dist_P =  7 
cell_cycle_dist_loc_P = 0.8 * mean_cell_cycle_P
cell_cycle_dist_scale_P = 0.05 * mean_cell_cycle_P


def draw_random_cell_cycle_length(celltype):
    if celltype == 'P':
        cycle = gamma.rvs(a     = cell_cycle_dist_A,
                          loc   = cell_cycle_dist_loc_A,
                          scale = cell_cycle_dist_scale_A)
    elif celltype == 'G':
        cycle = gamma.rvs(a     = cell_cycle_dist_P,
                          loc   = cell_cycle_dist_loc_P,
                          scale = cell_cycle_dist_scale_P)
    elif celltype == 'B':
        cycle = np.nan
        
    return cycle

# Plotting the division distributions given a drawing area ax3
ax4 = plot_division_distributions(cell_cycle_dist_A, cell_cycle_dist_loc_A, cell_cycle_dist_scale_A, cell_cycle_dist_P, cell_cycle_dist_loc_P, cell_cycle_dist_scale_P, ax4)
fig_model.show()
```

```
/usr/lib/python3.6/site-packages/matplotlib/figure.py:402: UserWarning: matplotlib is currently using a non-GUI backend, so cannot show the figure
  "matplotlib is currently using a non-GUI backend, "
```

## State transitions (c,q,model)¶

In [7]:

```
def state_transitions(c,q,model):
    #States = ['B', 'G', 'P'] # BLUE GREEN PURPLE

    if model == 'sine':
        f = lambda t, q: blue_probab_sine(t,q)
    elif model == 'line':
        f = lambda t, q: blue_probab_line(t,q)
    elif model == 'flat':
        f = lambda t, q: blue_probab_flat(t,q)

    BB = 1;                     BG = 0;       BP = 0
    GB = lambda t: c*f(t,q);    GG = 1-c;     GP = lambda t: c*(1-f(t,q))
    PB = 0;                     PG = 0;       PP = 1

    
    cumsum_list = lambda t: [GG, GG+GB(t),1]
    STdict = {'BB':BB,'BG':BG,'BP':BP,
              'GB':GB,'GG':GG,'GP':GP,
              'PB':PB,'PG':PG,'PP':PP,}
    
    return STdict
```

## Get offspring¶

In [8]:

```
def get_offspring(cell_type,STdict,time):
    rand = random()
    # # # Celltype G # # #
    if cell_type == 'G':
        if rand < STdict['GG']:         # This it the chance that it will Be G-->G
            return 'G'
        elif rand < STdict['GG']+STdict['GB'](time): # If it did not go to G, but is still below GG+GB(t), then it must be in a range of size GB(t), so this is the G->B
            return 'B'
        else:                 # if it did not go to any of the others, then because to probabs sum to 1, it must be G->P
            return 'P'
    
    # # # Celltype P # # #   
    elif cell_type == 'P':
        if rand < STdict['PG']:         # This it the chance that it will Be P -->G
            return 'G'
        elif rand < STdict['PG']+STdict['PB']: # If it did not go to G, but is still below PG+PB, then it must be in a range of size PB, so this is the P->B
            return 'B'
        else:                 # if it did not go to any of the others, then because to probabs sum to 1, it must be P->P
            return 'P'
    
    # # # Celltype B # # #
    elif cell_type == 'B':
        if rand < STdict['BG']:         # This it the chance that it will Be B-->G
            return 'G'
        elif rand < STdict['BG']+STdict['BB']: # If it did not go to G, but is still below BG+BB, then it must be in a range of size BB, so this is the B->B
            return 'B'
        else:                 # if it did not go to any of the others, then because to probabs sum to 1, it must be B->P
            return 'P'
```

# Make the cells divide¶

## Simulation code¶

In [9]:

```
# Simulation code
def time_convert_228h_348h_to_0_1(input_in_hours): # 348 is 14.5, 228 is 9.5
    return (input_in_hours - 228)/(348-228)

def cell_sim_make_init_condition_v_0_1(c,q,model,n_max_cells, starttime = 9.5, starttype = 'G'):
    cd = {0: [0,dict()]} # CellDict
    n = n_max_cells
    
    init_cells = pd.DataFrame({ 'DICT'             :            [{} for i in range(n)],
                                'generation'       : np.array(  n * [0 ]),
                                'next_divide_time' : np.array(  n * [np.nan]),
                                'offspring'        :            n * [[] ],
                                'parent'           : np.array(  n * [np.nan]),
                                'time_born'        : np.array(  n * [0 ]),
                                'times_divided'    : np.array(  n * [0 ]),
                                'type'             : n * ['NON'],
                              })
    #### List init cells here ####
    ndt = draw_random_cell_cycle_length
    ################  [ 'DICT'  , 'generation', 'next_divide_time'         , 'offspring', 'parent', 'time_born', 'times_divided', 'type']
    init_cells.loc[0]=[ cd[0][1],       0     ,  starttime*24 + random()*ndt('G'),      []    ,  np.nan ,    starttime*24  ,       0        ,   starttype]
    #init_cells.loc[1]=[ cd[1][1],       0    ,         0                  ,      []    ,  np.nan ,       0    ,       0        ,   'G']
    #init_cells.loc[2]=[ cd[2][1],       0    ,         0                  ,      []    ,  np.nan ,       0    ,       0        ,   'G']
    n_cells = 1
    return init_cells, cd, n_cells

def cell_sim(init_cells, cd, c, q, model,timestop,n_cells):
    STdict = state_transitions(c,q,model)
    cells = deepcopy(init_cells)
    n_cells = n_cells #len(init_cells)
    time_divided = 0
    recorder_counter = 0
    time_to_record = [10.5, 11.5, 12.5, 14.5]
    recorded_P = []
    recorded_B = []
    recorded_G = []
    recorded_total = []
    time_to_record = time_to_record + [100000, 200000, 300000, 400000] # ARBITRARY HIGH NUMBERS
    time_to_record = time_to_record
    
    
    while time_divided < timestop * 24: # 14.5 * 24:
        idx_to_divide = cells['next_divide_time'].idxmin(skipna = True)
        if np.isnan(idx_to_divide):
            while len(recorded_P) < 4:
                recorded_P.append(deepcopy(sum(cells['type'] == 'P')))
                recorded_G.append(deepcopy(sum(cells['type'] == 'G')))
                recorded_B.append(deepcopy(sum(cells['type'] == 'B')))
                recorded_total.append(recorded_P[-1]+recorded_G[-1]+recorded_B[-1])
                recorder_counter += 1
            break
            
        type_to_divide = cells['type'][idx_to_divide]
        time_divided = cells['next_divide_time'][idx_to_divide]
        
        #recorder
        while time_divided > 24*time_to_record[recorder_counter]:
            recorded_P.append(deepcopy(sum(cells['type'] == 'P')))
            recorded_G.append(deepcopy(sum(cells['type'] == 'G')))
            recorded_B.append(deepcopy(sum(cells['type'] == 'B')))
            recorded_total.append(recorded_P[-1]+recorded_G[-1]+recorded_B[-1])
            recorder_counter += 1
        
        parent_idx = idx_to_divide
        offspring_idx = n_cells  # Since python 0 indexes, the idx of the new cell should be same as n_current_cells

        time = time_convert_228h_348h_to_0_1(time_divided)
        
        # Create offspring
        offspring_generation = cells['generation'][parent_idx] + 1
        offspring_type = get_offspring(type_to_divide,STdict,time)
        offspring_times_divided = 0 # Obviously
        offsprint_parent = parent_idx
        offspring_time_born = time_divided
        offspring_next_divide_time = offspring_time_born + draw_random_cell_cycle_length(offspring_type)

        cells.loc[offspring_idx]=[{},offspring_generation, offspring_next_divide_time, [], offsprint_parent, offspring_time_born, offspring_times_divided, offspring_type] 

        # Update parent cell
        parents_new_type = get_offspring(type_to_divide,STdict,time)
        cells.loc[parent_idx,'type'] = parents_new_type
        cells.loc[parent_idx,'next_divide_time'] = time_divided + draw_random_cell_cycle_length(parents_new_type)
        cells.loc[parent_idx,'times_divided'] += 1
        cells.loc[parent_idx,'offspring'].append(offspring_idx)

#         # Make CellDict for heritage plotting
#         cells.loc[parent_idx,'DICT'][parent_idx]    = [1, offspring_time_born, {}]
#         cells.loc[parent_idx,'DICT'][offspring_idx] = [1, offspring_time_born, {}]
#         cells.set_value(offspring_idx, 'DICT', cells.loc[parent_idx,'DICT'][offspring_idx][2]) 
#         cells.set_value(parent_idx, 'DICT', cells.loc[parent_idx,'DICT'][parent_idx][2])
        
        n_cells += 1
    return cells, cd, n_cells, recorded_P, recorded_G, recorded_B, recorded_total

def run_cell_sim(c,q,model,n_max_cells,timestop, starttime = 9.5, starttype = 'G'):
    init_cells, cd = cell_sim_make_init_condition_v_0_1(c,q,model,n_max_cells, starttime = starttime, starttype = starttype)
    simu_cells, cd, n_cells = cell_sim(init_cells,cd,c,q,model,timestop)
    decendant_info = get_decendants_numbers(simu_cells)
    return simu_cells[0:n_cells], cd, decendant_info, n_cells

def run_cell_sim(c,q,model,n_max_cells,timestop, starttime = 9.5, starttype = 'G'):
    init_cells, cd, n_cells = cell_sim_make_init_condition_v_0_1(c,q,model,n_max_cells, starttime = starttime, starttype = starttype)
    simu_cells, cd, n_cells, recorded_P, recorded_G, recorded_B, recorded_total = cell_sim(init_cells,cd,c,q,model,timestop, n_cells = n_cells)
    decendant_info = get_decendants_numbers(simu_cells)
    return simu_cells[0:n_cells], cd, decendant_info, n_cells, recorded_P, recorded_G, recorded_B, recorded_total
```

## Initial conditions¶

## Run simulation¶

In [67]:

```
init_time = 0
c = 0.15
q = 0.37
model = 'sine'
n_max_cells = 1
starttime = 9.5
timestop = 11.5


#simu_cells, cd, decendant_info, n_cells = run_cell_sim(c,q,model,n_max_cells,timestop)
init_cells, cd, n_cells = cell_sim_make_init_condition_v_0_1(c,q,model,n_max_cells = 100, starttime = starttime, starttype = 'G')
simu_cells, cd, n_cells, recorded_P, recorded_G, recorded_B, recorded_total = cell_sim(init_cells,cd,c,q,model,timestop, n_cells = 1)
print(recorded_P, recorded_G, recorded_B, recorded_total)
```

```
[0, 0] [3, 6] [1, 1] [4, 7]
```

In [68]:

```
simu_cells['time_born']/24
```

Out[68]:

```
0      9.500000
1      9.501404
2     10.276876
3     10.310008
4     11.001743
5     11.010260
6     11.201961
7     11.631389
8      0.000000
9      0.000000
10     0.000000
11     0.000000
12     0.000000
13     0.000000
14     0.000000
15     0.000000
16     0.000000
17     0.000000
18     0.000000
19     0.000000
20     0.000000
21     0.000000
22     0.000000
23     0.000000
24     0.000000
25     0.000000
26     0.000000
27     0.000000
28     0.000000
29     0.000000
        ...    
70     0.000000
71     0.000000
72     0.000000
73     0.000000
74     0.000000
75     0.000000
76     0.000000
77     0.000000
78     0.000000
79     0.000000
80     0.000000
81     0.000000
82     0.000000
83     0.000000
84     0.000000
85     0.000000
86     0.000000
87     0.000000
88     0.000000
89     0.000000
90     0.000000
91     0.000000
92     0.000000
93     0.000000
94     0.000000
95     0.000000
96     0.000000
97     0.000000
98     0.000000
99     0.000000
Name: time_born, dtype: float64
```

In [69]:

```
simu_cells
```

Out[69]:

|  | DICT | generation | next\_divide\_time | offspring | parent | time\_born | times\_divided | type |
| --- | --- | --- | --- | --- | --- | --- | --- | --- |
| 0 | {} | 0 | NaN | [1, 2] | NaN | 228.000000 | 2 | B |
| 1 | {} | 1 | 282.320729 | [3, 5] | 0.0 | 228.033708 | 2 | G |
| 2 | {} | 1 | 283.745821 | [6] | 0.0 | 246.645031 | 1 | G |
| 3 | {} | 2 | 279.994679 | [4] | 1.0 | 247.440184 | 1 | G |
| 4 | {} | 3 | 297.371420 | [7] | 3.0 | 264.041841 | 1 | G |
| 5 | {} | 2 | 281.110511 | [] | 1.0 | 264.246240 | 0 | G |
| 6 | {} | 2 | 287.585418 | [] | 2.0 | 268.847064 | 0 | G |
| 7 | {} | 4 | NaN | [] | 4.0 | 279.153326 | 0 | B |
| 8 | {} | 0 | NaN | [] | NaN | 0.000000 | 0 | NON |
| 9 | {} | 0 | NaN | [] | NaN | 0.000000 | 0 | NON |
| 10 | {} | 0 | NaN | [] | NaN | 0.000000 | 0 | NON |
| 11 | {} | 0 | NaN | [] | NaN | 0.000000 | 0 | NON |
| 12 | {} | 0 | NaN | [] | NaN | 0.000000 | 0 | NON |
| 13 | {} | 0 | NaN | [] | NaN | 0.000000 | 0 | NON |
| 14 | {} | 0 | NaN | [] | NaN | 0.000000 | 0 | NON |
| 15 | {} | 0 | NaN | [] | NaN | 0.000000 | 0 | NON |
| 16 | {} | 0 | NaN | [] | NaN | 0.000000 | 0 | NON |
| 17 | {} | 0 | NaN | [] | NaN | 0.000000 | 0 | NON |
| 18 | {} | 0 | NaN | [] | NaN | 0.000000 | 0 | NON |
| 19 | {} | 0 | NaN | [] | NaN | 0.000000 | 0 | NON |
| 20 | {} | 0 | NaN | [] | NaN | 0.000000 | 0 | NON |
| 21 | {} | 0 | NaN | [] | NaN | 0.000000 | 0 | NON |
| 22 | {} | 0 | NaN | [] | NaN | 0.000000 | 0 | NON |
| 23 | {} | 0 | NaN | [] | NaN | 0.000000 | 0 | NON |
| 24 | {} | 0 | NaN | [] | NaN | 0.000000 | 0 | NON |
| 25 | {} | 0 | NaN | [] | NaN | 0.000000 | 0 | NON |
| 26 | {} | 0 | NaN | [] | NaN | 0.000000 | 0 | NON |
| 27 | {} | 0 | NaN | [] | NaN | 0.000000 | 0 | NON |
| 28 | {} | 0 | NaN | [] | NaN | 0.000000 | 0 | NON |
| 29 | {} | 0 | NaN | [] | NaN | 0.000000 | 0 | NON |
| ... | ... | ... | ... | ... | ... | ... | ... | ... |
| 70 | {} | 0 | NaN | [] | NaN | 0.000000 | 0 | NON |
| 71 | {} | 0 | NaN | [] | NaN | 0.000000 | 0 | NON |
| 72 | {} | 0 | NaN | [] | NaN | 0.000000 | 0 | NON |
| 73 | {} | 0 | NaN | [] | NaN | 0.000000 | 0 | NON |
| 74 | {} | 0 | NaN | [] | NaN | 0.000000 | 0 | NON |
| 75 | {} | 0 | NaN | [] | NaN | 0.000000 | 0 | NON |
| 76 | {} | 0 | NaN | [] | NaN | 0.000000 | 0 | NON |
| 77 | {} | 0 | NaN | [] | NaN | 0.000000 | 0 | NON |
| 78 | {} | 0 | NaN | [] | NaN | 0.000000 | 0 | NON |
| 79 | {} | 0 | NaN | [] | NaN | 0.000000 | 0 | NON |
| 80 | {} | 0 | NaN | [] | NaN | 0.000000 | 0 | NON |
| 81 | {} | 0 | NaN | [] | NaN | 0.000000 | 0 | NON |
| 82 | {} | 0 | NaN | [] | NaN | 0.000000 | 0 | NON |
| 83 | {} | 0 | NaN | [] | NaN | 0.000000 | 0 | NON |
| 84 | {} | 0 | NaN | [] | NaN | 0.000000 | 0 | NON |
| 85 | {} | 0 | NaN | [] | NaN | 0.000000 | 0 | NON |
| 86 | {} | 0 | NaN | [] | NaN | 0.000000 | 0 | NON |
| 87 | {} | 0 | NaN | [] | NaN | 0.000000 | 0 | NON |
| 88 | {} | 0 | NaN | [] | NaN | 0.000000 | 0 | NON |
| 89 | {} | 0 | NaN | [] | NaN | 0.000000 | 0 | NON |
| 90 | {} | 0 | NaN | [] | NaN | 0.000000 | 0 | NON |
| 91 | {} | 0 | NaN | [] | NaN | 0.000000 | 0 | NON |
| 92 | {} | 0 | NaN | [] | NaN | 0.000000 | 0 | NON |
| 93 | {} | 0 | NaN | [] | NaN | 0.000000 | 0 | NON |
| 94 | {} | 0 | NaN | [] | NaN | 0.000000 | 0 | NON |
| 95 | {} | 0 | NaN | [] | NaN | 0.000000 | 0 | NON |
| 96 | {} | 0 | NaN | [] | NaN | 0.000000 | 0 | NON |
| 97 | {} | 0 | NaN | [] | NaN | 0.000000 | 0 | NON |
| 98 | {} | 0 | NaN | [] | NaN | 0.000000 | 0 | NON |
| 99 | {} | 0 | NaN | [] | NaN | 0.000000 | 0 | NON |

100 rows × 8 columns

In [57]:

```
pprint(len(simu_cells))
pprint(n_cells)
simu_cells['type'].value_counts()
#pprint(cd)
simu_cells
```

```
100
3
```

Out[57]:

|  | DICT | generation | next\_divide\_time | offspring | parent | time\_born | times\_divided | type |
| --- | --- | --- | --- | --- | --- | --- | --- | --- |
| 0 | {} | 0 | 264.500735 | [1] | NaN | 228.000000 | 1 | G |
| 1 | {} | 1 | 279.190099 | [2] | 0.0 | 245.593506 | 1 | P |
| 2 | {} | 2 | 280.591515 | [] | 1.0 | 262.084940 | 0 | G |
| 3 | {} | 0 | NaN | [] | NaN | 0.000000 | 0 | NON |
| 4 | {} | 0 | NaN | [] | NaN | 0.000000 | 0 | NON |
| 5 | {} | 0 | NaN | [] | NaN | 0.000000 | 0 | NON |
| 6 | {} | 0 | NaN | [] | NaN | 0.000000 | 0 | NON |
| 7 | {} | 0 | NaN | [] | NaN | 0.000000 | 0 | NON |
| 8 | {} | 0 | NaN | [] | NaN | 0.000000 | 0 | NON |
| 9 | {} | 0 | NaN | [] | NaN | 0.000000 | 0 | NON |
| 10 | {} | 0 | NaN | [] | NaN | 0.000000 | 0 | NON |
| 11 | {} | 0 | NaN | [] | NaN | 0.000000 | 0 | NON |
| 12 | {} | 0 | NaN | [] | NaN | 0.000000 | 0 | NON |
| 13 | {} | 0 | NaN | [] | NaN | 0.000000 | 0 | NON |
| 14 | {} | 0 | NaN | [] | NaN | 0.000000 | 0 | NON |
| 15 | {} | 0 | NaN | [] | NaN | 0.000000 | 0 | NON |
| 16 | {} | 0 | NaN | [] | NaN | 0.000000 | 0 | NON |
| 17 | {} | 0 | NaN | [] | NaN | 0.000000 | 0 | NON |
| 18 | {} | 0 | NaN | [] | NaN | 0.000000 | 0 | NON |
| 19 | {} | 0 | NaN | [] | NaN | 0.000000 | 0 | NON |
| 20 | {} | 0 | NaN | [] | NaN | 0.000000 | 0 | NON |
| 21 | {} | 0 | NaN | [] | NaN | 0.000000 | 0 | NON |
| 22 | {} | 0 | NaN | [] | NaN | 0.000000 | 0 | NON |
| 23 | {} | 0 | NaN | [] | NaN | 0.000000 | 0 | NON |
| 24 | {} | 0 | NaN | [] | NaN | 0.000000 | 0 | NON |
| 25 | {} | 0 | NaN | [] | NaN | 0.000000 | 0 | NON |
| 26 | {} | 0 | NaN | [] | NaN | 0.000000 | 0 | NON |
| 27 | {} | 0 | NaN | [] | NaN | 0.000000 | 0 | NON |
| 28 | {} | 0 | NaN | [] | NaN | 0.000000 | 0 | NON |
| 29 | {} | 0 | NaN | [] | NaN | 0.000000 | 0 | NON |
| ... | ... | ... | ... | ... | ... | ... | ... | ... |
| 70 | {} | 0 | NaN | [] | NaN | 0.000000 | 0 | NON |
| 71 | {} | 0 | NaN | [] | NaN | 0.000000 | 0 | NON |
| 72 | {} | 0 | NaN | [] | NaN | 0.000000 | 0 | NON |
| 73 | {} | 0 | NaN | [] | NaN | 0.000000 | 0 | NON |
| 74 | {} | 0 | NaN | [] | NaN | 0.000000 | 0 | NON |
| 75 | {} | 0 | NaN | [] | NaN | 0.000000 | 0 | NON |
| 76 | {} | 0 | NaN | [] | NaN | 0.000000 | 0 | NON |
| 77 | {} | 0 | NaN | [] | NaN | 0.000000 | 0 | NON |
| 78 | {} | 0 | NaN | [] | NaN | 0.000000 | 0 | NON |
| 79 | {} | 0 | NaN | [] | NaN | 0.000000 | 0 | NON |
| 80 | {} | 0 | NaN | [] | NaN | 0.000000 | 0 | NON |
| 81 | {} | 0 | NaN | [] | NaN | 0.000000 | 0 | NON |
| 82 | {} | 0 | NaN | [] | NaN | 0.000000 | 0 | NON |
| 83 | {} | 0 | NaN | [] | NaN | 0.000000 | 0 | NON |
| 84 | {} | 0 | NaN | [] | NaN | 0.000000 | 0 | NON |
| 85 | {} | 0 | NaN | [] | NaN | 0.000000 | 0 | NON |
| 86 | {} | 0 | NaN | [] | NaN | 0.000000 | 0 | NON |
| 87 | {} | 0 | NaN | [] | NaN | 0.000000 | 0 | NON |
| 88 | {} | 0 | NaN | [] | NaN | 0.000000 | 0 | NON |
| 89 | {} | 0 | NaN | [] | NaN | 0.000000 | 0 | NON |
| 90 | {} | 0 | NaN | [] | NaN | 0.000000 | 0 | NON |
| 91 | {} | 0 | NaN | [] | NaN | 0.000000 | 0 | NON |
| 92 | {} | 0 | NaN | [] | NaN | 0.000000 | 0 | NON |
| 93 | {} | 0 | NaN | [] | NaN | 0.000000 | 0 | NON |
| 94 | {} | 0 | NaN | [] | NaN | 0.000000 | 0 | NON |
| 95 | {} | 0 | NaN | [] | NaN | 0.000000 | 0 | NON |
| 96 | {} | 0 | NaN | [] | NaN | 0.000000 | 0 | NON |
| 97 | {} | 0 | NaN | [] | NaN | 0.000000 | 0 | NON |
| 98 | {} | 0 | NaN | [] | NaN | 0.000000 | 0 | NON |
| 99 | {} | 0 | NaN | [] | NaN | 0.000000 | 0 | NON |

100 rows × 8 columns

## Run N simulations¶

In [53]:

```
init_time = 0
c = 0.08 # How much of G goes into B and P
q = 0.22
model = 'flat'
n_max_cells = 100
timestop = 14.5

cell_count_total = []
cell_count_P = []
cell_count_G = []
cell_count_B = []
for i in tqdm(range(30)):
    #simu_cells, cd, decendant_info, n_cells = run_cell_sim(c,q,model,200,timestop, starttime = 9.5, starttype = 'G')
    init_cells    , cd, n_cells = cell_sim_make_init_condition_v_0_1(c,q,model,1, starttime =11.5, starttype = 'G')
    #simu_cells_105, cd, n_cells, recorded_P, recorded_G, recorded_B, recorded_total = cell_sim(init_cells    ,cd,c,q,model,timestop = 10.5, n_cells = n_cells)
    #simu_cells_115, cd, n_cells, recorded_P, recorded_G, recorded_B, recorded_total = cell_sim(simu_cells_105,cd,c,q,model,timestop = 11.5, n_cells = n_cells)
    #simu_cells_125, cd, n_cells, recorded_P, recorded_G, recorded_B, recorded_total = cell_sim(simu_cells_115,cd,c,q,model,timestop = 12.5, n_cells = n_cells)
    #simu_cells    , cd, n_cells, recorded_P, recorded_G, recorded_B, recorded_total = cell_sim(simu_cells_115,cd,c,q,model,timestop = 14.5, n_cells = n_cells)
    simu_cells    , cd, n_cells, recorded_P, recorded_G, recorded_B, recorded_total = cell_sim(init_cells,cd,c,q,model,timestop = 14.5, n_cells = n_cells)

    
    cell_count_total.append(len(simu_cells))
    cell_count_P.append(sum(simu_cells['type'] == 'P'))
    cell_count_G.append(sum(simu_cells['type'] == 'G'))
    cell_count_B.append(sum(simu_cells['type'] == 'B'))
    
cell_count_total2 = []
cell_count_P2 = []
cell_count_G2 = []
cell_count_B2 = []
for i in tqdm(range(24)):
    simu_cells, cd, decendant_info, n_cells, recorded_P, recorded_G, recorded_B, recorded_total = run_cell_sim(c,q,model,200,timestop, starttime = 11.5, starttype = 'G')
#     init_cells    , cd, n_cells = cell_sim_make_init_condition_v_0_1(c,q,model,1, starttime = 9.5, starttype = 'G')
#     simu_cells_105, cd, n_cells = cell_sim(init_cells    ,cd,c,q,model,timestop = 10.5, n_cells = n_cells)
#     simu_cells_115, cd, n_cells = cell_sim(simu_cells_105,cd,c,q,model,timestop = 11.5, n_cells = n_cells)
#     simu_cells_125, cd, n_cells = cell_sim(simu_cells_115,cd,c,q,model,timestop = 12.5, n_cells = n_cells)
#     simu_cells    , cd, n_cells = cell_sim(simu_cells_115,cd,c,q,model,timestop = 14.5, n_cells = n_cells)

    cell_count_total2.append(len(simu_cells))
    cell_count_P2.append(sum(simu_cells['type'] == 'P'))
    cell_count_G2.append(sum(simu_cells['type'] == 'G'))
    cell_count_B2.append(sum(simu_cells['type'] == 'B'))
    
if 1 == 1:
## Nice bar
    % matplotlib notebook
    b_for_all, g_for_all, p_for_all, totals, idx_1, idx_2, idx_3 = sort_and_group(cell_count_total, cell_count_B, cell_count_G, cell_count_P)
    fig1, ax1 = plot_sort_and_group(b_for_all, g_for_all, p_for_all, totals, idx_1, idx_2, idx_3)
    fig1.show()

    b_for_all, g_for_all, p_for_all, totals, idx_1, idx_2, idx_3 = sort_and_group(cell_count_total2, cell_count_B2, cell_count_G2, cell_count_P2)
    fig2, ax1 = plot_sort_and_group(b_for_all, g_for_all, p_for_all, totals, idx_1, idx_2, idx_3)
    fig2.show()
```

```
100%|██████████| 30/30 [00:02<00:00, 11.70it/s]
100%|██████████| 24/24 [00:01<00:00, 16.71it/s]
```

In [ ]:

```

```

In [39]:

```
#fig = plt.gcf()
fig1.savefig('z_115-145_3.svg')
```

### Ratio dist¶

In [1382]:

```
#group_2_b_ratio = b_for_all[idx_2] / totals[idx_2]
#group_3_p_ratio = p_for_all[idx_3] / totals[idx_3]
```

In [1383]:

```
# fig = plt.figure()
# n, bins, patches = plt.hist(group_3_p_ratio, bins=10, range=[0,1], align='mid')
# fig.show()
```

# Parameter scan¶

## Set parameter range¶

In [1501]:

```
c_index = np.linspace(0,0.4,20)
q_index = np.linspace(0,1,20)
sims_per_paramset = 500
model_defined = 'sine'

c_index = c_index[1:]
q_index = q_index[0:-1]
num_total_param_configs = len(c_index)*len(q_index)*sims_per_paramset

print('simulation_5'+'-'+model_defined)
data_name =   'paramscan_data_df_c0to04_'+model_defined+'_withfixedtimes_1.json'
times_name = 'paramscan_times_df_c0to04_'+model_defined+'_withfixedtimes_1.json'
print(data_name)
print(times_name)
```

```
simulation_5-sine
paramscan_data_df_c0to04_sine_withfixedtimes_1.json
paramscan_times_df_c0to04_sine_withfixedtimes_1.json
```

## Parameter scan¶

In [1484]:

```
paramscan_data = defaultdict(lambda: defaultdict(lambda: {}))
paramscan_times = defaultdict(lambda: defaultdict(lambda: {}))

with tqdm(total=num_total_param_configs) as pbar:
    for c_i in (c_index):
        for q_i in (q_index):
            c = c_i
            q = q_i
            model = model_defined
            n_max_cells = 200
            timestop = 14.5

            cell_count_total = []; cell_count_P = []; cell_count_G = []; cell_count_B = []
            B_at_times_holder = []; P_at_times_holder = []; G_at_times_holder = []; Total_at_times_holder = [];
            
            for i in range(sims_per_paramset):

                simu_cells, cd, decendant_info, n_cells, recorded_P, recorded_G, recorded_B,recorded_total = run_cell_sim(c,q,model,n_max_cells,timestop)

                cell_count_total.append(len(simu_cells))
                cell_count_P.append(sum(simu_cells['type'] == 'P'))
                cell_count_G.append(sum(simu_cells['type'] == 'G'))
                cell_count_B.append(sum(simu_cells['type'] == 'B'))

                times = [9.5*24, 10.5*24, 11.5*24, 12.5*24, 14.5*24]
                B_at_times     = []; P_at_times = []; G_at_times = []; Total_at_times = [];
                for n in range(4):
                    time1 = times[n]; time2 = times[n+1]
                    B_at_times.append(  recorded_B[n]  )
                    P_at_times.append(  recorded_P[n]  )
                    G_at_times.append(  recorded_G[n]  )
                    Total_at_times.append(  recorded_total[n]  )

                B_at_times_holder.append(B_at_times)
                P_at_times_holder.append(P_at_times)
                G_at_times_holder.append(G_at_times)
                Total_at_times_holder.append(Total_at_times)
                
                pbar.update()
            
            paramscan_data[str(c)][str(q)]['total'] = cell_count_total
            paramscan_data[str(c)][str(q)]['P'] = cell_count_P 
            paramscan_data[str(c)][str(q)]['G'] = cell_count_G
            paramscan_data[str(c)][str(q)]['B'] = cell_count_B
            paramscan_times[str(c)][str(q)]['P_times'] = P_at_times_holder
            paramscan_times[str(c)][str(q)]['G_times'] = G_at_times_holder
            paramscan_times[str(c)][str(q)]['B_times'] = B_at_times_holder
            paramscan_times[str(c)][str(q)]['total_times'] = Total_at_times_holder
            
paramscan_data_df = pd.DataFrame.from_dict(paramscan_data)
paramscan_times_df = pd.DataFrame.from_dict(paramscan_times)

# Save the data
paramscan_data_df.to_json(data_name)
paramscan_times_df.to_json(times_name)
```

```
 95%|█████████▌| 760/800 [02:42<00:02, 14.92it/s]
```

In [1491]:

```
paramscan_times_df.iloc[5,5]
```

Out[1491]:

```
{'B_times': [[0, 1, 1, 13], [1, 1, 1, 12]],
 'G_times': [[3, 6, 13, 65], [1, 3, 8, 38]],
 'P_times': [[0, 1, 4, 30], [0, 0, 0, 7]],
 'total_times': [[3, 8, 18, 108], [2, 4, 9, 57]]}
```

In [1487]:

```
# #paramscan_data_df.to_json('paramscan_data_df_16-02.json')
# c_index = np.linspace(0,1,20)
# q_index = np.linspace(0,1,20)
# paramscan_data_df = pd.read_json('paramscan_data_df_16-02.json')
# # fix
# paramscan_data_df = paramscan_data_df.set_index(c_index)
# paramscan_data_df.columns = q_index

#paramscan_data_df.to_json('paramscan_data_df_16-02.json')
# c_index = np.linspace(0,0.4,20)
# q_index = np.linspace(0,1  ,20)
# paramscan_data_df = pd.read_json('paramscan_data_df_c0to04.json')
# # fix
# paramscan_data_df.index = q_index
# paramscan_data_df.columns = c_index
```

In [ ]:

```

```

In [ ]:

```

```

In [ ]:

```

```
