# Supplementary material for: Stochastic priming and spatial cues orchestrate heterogeneous clonal contribution to mouse pancreas organogenesis
Source: Nat Commun. 2017 Sep 19;8:605. doi: 10.1038/s41467-017-00258-4 (PMC5605525; doi:10.1038/s41467-017-00258-4)
Supplement: Supplementary file 2 — Supplementary Software [file 41467_2017_258_MOESM2_ESM.zip › code/Analyze_5.html]

Analyze\_5


# Import statements¶

In [1]:

```
# init
import os, sys
sys.path.insert(0,os.path.pardir)
import pandas as pd

from scipy import stats
import collections
import matplotlib.pylab as plt
from matplotlib import interactive
interactive(False)

import glob
import scipy.signal
import json

#import mpld3
#mpld3.enable_notebook()

import numpy as np
from pprint import pprint
import scipy
import scipy.special
import scipy.stats
import random
from collections import defaultdict
from copy import copy, deepcopy
import colorama
from colorama import Fore, Back, Style

from scipy.stats import norm
from sklearn.neighbors import KernelDensity

from tqdm import tqdm, tqdm_notebook, tnrange
from statsmodels.distributions.empirical_distribution import ECDF

%load_ext line_profiler
%matplotlib notebook

from scipy.interpolate import interp1d
from scipy.stats import norm, gamma

figwidth = 10
figheight = 5

plot = True
```

# Misc Func definitions¶

In [2]:

```
# Visualize f(t) models given two plotting areas ax1 and ax2
def plot_f_of_t(blue_probab_sine, purple_probab_sine,blue_probab_line, purple_probab_line, blue_probab_flat, purple_probab_flat, ax1, ax2, ax3, q = 0.2):
    q = q
    start_height_blue = 1; min_height_blue = q
    shb = start_height_blue; mhb = min_height_blue
    arrowheight = 0.03
    t = np.linspace(0,1,101)
    
    ax1.set_title('model 1')
    ax1.clear()
    ax1.fill_between(t, blue_probab_sine(t,mhb) , alpha = 0.5, color = 'blue'  , label = 'G -> B, ($f$ )')
    ax1.fill_between(t, purple_probab_sine(t,mhb), alpha = 0.5, color = 'purple', label = 'G -> P, ($1-f$ )')
    ax1.set_xlabel('time'); ax1.set_ylabel('fraction'); ax1.legend()

    ax1.arrow(0.5, 0,   0, mhb -arrowheight, head_width=0.03, head_length=arrowheight, fc='k', ec='k')
    ax1.arrow(0.5, mhb, 0, -mhb+arrowheight, head_width=0.03, head_length=arrowheight, fc='k', ec='k')
    ax1.text(0.52, mhb/2-0.02, r'$q$', fontsize=15)

    ax2.set_title('model 2'); 
    ax2.clear()
    ax2.fill_between(t,  blue_probab_line(t,mhb) , alpha = 0.5, color = 'blue'  , label = 'G -> B, ($f$ )')
    ax2.fill_between(t,purple_probab_line(t,mhb), alpha = 0.5, color = 'purple', label = 'G -> P, ($1-f$ )')
    ax2.set_xlabel('time'); ax2.set_ylabel('fraction'); ax2.legend()

    ax2.arrow(0.5, 0,   0, mhb -arrowheight, head_width=0.03, head_length=arrowheight, fc='k', ec='k')
    ax2.arrow(0.5, mhb, 0, -mhb+arrowheight, head_width=0.03, head_length=arrowheight, fc='k', ec='k')
    ax2.text(0.52, mhb/2-0.02, r'$q$', fontsize=15)
                     
    ax3.set_title('model 3'); 
    ax3.clear()
    ax3.fill_between(t,  blue_probab_flat(t,mhb) , alpha = 0.5, color = 'blue'  , label = 'G -> B, ($f$ )')
    ax3.fill_between(t,purple_probab_flat(t,mhb), alpha = 0.5, color = 'purple', label = 'G -> P, ($1-f$ )')
    ax3.set_xlabel('time'); ax3.set_ylabel('fraction'); ax3.legend()

    ax3.arrow(0.5, 0,   0, mhb -arrowheight, head_width=0.03, head_length=arrowheight, fc='k', ec='k')
    ax3.arrow(0.5, mhb, 0, -mhb+arrowheight, head_width=0.03, head_length=arrowheight, fc='k', ec='k')
    ax3.text(0.52, mhb/2-0.02, r'$q$', fontsize=15)
    return ax1, ax2, ax3

# Plotting the division distributions given a drawing area ax3
def plot_division_distributions(cell_cycle_dist_A, cell_cycle_dist_loc_A, cell_cycle_dist_scale_A, cell_cycle_dist_P, cell_cycle_dist_loc_P, cell_cycle_dist_scale_P, ax3):
    ax3.set_title('Distribution');
    ax3.clear()
    t_n = np.linspace(0,100,1000)

    # Plot the actual distribution
    if 1 == 1:
        ax3.plot(t_n, scipy.stats.gamma.pdf(t_n,
                                            a     = cell_cycle_dist_A,
                                            loc   = cell_cycle_dist_loc_A,
                                            scale = cell_cycle_dist_scale_A), 
                 linewidth = 2, color = 'purple', label = 'purple')
        ax3.plot(t_n, scipy.stats.gamma.pdf(t_n,
                                            a     = cell_cycle_dist_P,
                                            loc   = cell_cycle_dist_loc_P,
                                            scale = cell_cycle_dist_scale_P), 
                 linewidth = 2, color = 'green' , label = 'green')
        ax3.plot(t_n, t_n*0, linewidth = 2, color = 'blue'  , label = 'blue')

    # Define rand-generator
    def green_dist(size = 1):
        return scipy.stats.gamma.rvs(a     = cell_cycle_dist_P,
                                     loc   = cell_cycle_dist_loc_P,
                                     scale = cell_cycle_dist_scale_P,
                                     size = size)
    def purple_dist(size = 1):
        return scipy.stats.gamma.rvs(a     = cell_cycle_dist_A,
                                     loc   = cell_cycle_dist_loc_A,
                                     scale = cell_cycle_dist_scale_A,
                                     size = size)

    # Make histogram of rand-generated nums - Green
    r_g = green_dist(size  = 1000);
    ax3.hist(r_g,bins = 50, normed=True, histtype='stepfilled', alpha=0.2, color = 'green');

    # Make histogram of rand-generated nums - Purple
    r_p = purple_dist(size=1000);
    ax3.hist(r_p,bins = 50, normed=True, histtype='stepfilled', alpha=0.2, color = 'purple');

    #plt.legend()
    ax3.set_title('Division distibution')
    ax3.set_xlabel('time [h]')
    ax3.set_ylabel('probability')
    ax3.set_xlim(5.0, 35.0);
    ax3.set_ylim(0.0,  0.3);
    return ax3

# Sorts all the results series of on parameter set and groups the clones of each simulation into 3 types
def sort_and_group(cell_count_total, cell_count_B, cell_count_G, cell_count_P):
    # Sort according to total
    idx=np.argsort(cell_count_total)
    # Make lists according to sort
    b_for_all = np.array(cell_count_B)[idx]
    g_for_all = np.array(cell_count_G)[idx]
    p_for_all = np.array(cell_count_P)[idx]
    totals = np.array(cell_count_total)[idx]
    # Logics for seperating into the groups.
    group3_bool = p_for_all != 0  # Find all the samples with acinar cells (These will have 3 types of cells)
    group1_bool = b_for_all == totals # Find all the samples that is only endocrine (Only one type of cell)
    group2_bool = (group3_bool | group1_bool) != 1 # Everything else. (This should be Progenitors and Endocrine) (2 types of cells)
    # Indicies for members of each of the groups
    idx_1 = np.array([num for num,elem in enumerate(group1_bool) if elem == True])
    idx_2 = np.array([num for num,elem in enumerate(group2_bool) if elem == True])
    idx_3 = np.array([num for num,elem in enumerate(group3_bool) if elem == True])
    return b_for_all, g_for_all, p_for_all, totals, idx_1, idx_2, idx_3

# Plots the above
def plot_sort_and_group(b_for_all, g_for_all, p_for_all, totals, idx_1, idx_2, idx_3, subplotlayout = 111, plotboxes = True, boxoffset = 0.4):
    num_clones = len(totals)
    r_c = np.arange(num_clones) # Range_Clones
    fig = plt.figure(figsize=(12,5))
    ax1 = fig.add_subplot(subplotlayout)

    # Plot the bars
    ax1.bar(r_c,b_for_all,                           color='#00BFFF',lw=0, label = 'B')
    ax1.bar(r_c,g_for_all,bottom=b_for_all,          color='#00FF00',lw=0, label = 'G')
    ax1.bar(r_c,p_for_all,bottom=b_for_all+g_for_all,color='#800080',lw=0, label = 'P')

    ax1.set_xlabel('Clone ID')
    ax1.set_ylabel('Clone size, cell #')

    if plotboxes == True:
        # Plot the boxes between the bars
        ax1.plot(idx_1+boxoffset,len(idx_1)*[totals.max() * -0.05],'bs')
        ax1.plot(idx_2+boxoffset,len(idx_2)*[totals.max() * -0.05],'gs')
        ax1.plot(idx_3+boxoffset,len(idx_3)*[totals.max() * -0.05],'rs');
        ax1.set_ylim([totals.max() * -0.1 , totals.max() * 1.1]);
    return fig, ax1

# Makes a dictionsary that incodes the information about decendants number and more. Recursive function!
def get_decendants_numbers(cells):
    n_decendants_dict = defaultdict(lambda: 1.0)
    def get_n_offspring(cell_n,cells,n_decendants_dict):
        list_of_offsprings = cells.loc[cell_n,'offspring']
        for number in list_of_offsprings:
            #n_decendants_dict[cell_n] = n_decendants_dict[cell_n] + 1.0
            n_decendants_dict[cell_n] = n_decendants_dict[cell_n] + get_n_offspring(number,cells,n_decendants_dict)
        if len(list_of_offsprings) == 0:
            return 1
        return n_decendants_dict[cell_n]
    get_n_offspring(0,cells,n_decendants_dict)
    return n_decendants_dict
```

In [ ]:

```

```

# Define and declare the system¶

## Intro text¶

The system can mostely be defined by a transition matrix. A transition matrix of two variables. $c$ and $f$

$$
\begin{bmatrix}
B \rightarrow B & B \rightarrow G & B \rightarrow P \\
G \rightarrow B & G \rightarrow G & G \rightarrow P \\
G \rightarrow B & G \rightarrow G & G \rightarrow P \\
\end{bmatrix}
=
\begin{bmatrix}
1 & 0 & 0 \\
c \cdot f & c & c\cdot(1-f) \\
0 & 0 & 1 \\
\end{bmatrix}
$$

Below is a diagram of the model. The distributions below each state represents cell divisions

The parameter controling the faction of green that goes to blue and purple, $f$, changes over time, while $c$ remains constant.

The change of $f$ is modeled according to the one of the two models below, where a new parameter is introduced. This parameter we call $q$.

So all in all there is 3 parameters; $f$,$c$ and $q$. It should be added here that $f$ is time dependand, $f(t)$, as per the model in use and the parameter $q$

In [3]:

```
if plot == True:
    fig_model = plt.figure('Model',figsize=(figwidth,figheight)); 
    ax1 = fig_model.add_subplot(1,4,1); ax2 = fig_model.add_subplot(1,4,2); ax3 = fig_model.add_subplot(1,4,3) ; ax4 = fig_model.add_subplot(1,4,4)
    fig_model.show()
```

## f(t)¶

In [4]:

```
#f(t)
# # # Sinus form # # #
blue_probab_sine =   lambda t,q: q+(1-q)*(1 - np.sin(t*np.pi)) 
blue_probab_sine =   lambda t,q: q+(1-q)*0.5*(1 + np.cos(t*2*np.pi))
purple_probab_sine = lambda t,q: 1-blue_probab_sine(t,q) # I do not really use these, since they are just 1-blue_probab
# # # Line form # # # 
blue_probab_line =   lambda t,q: q+(1-q)*(2*np.abs(0.5-t)) 
purple_probab_line = lambda t,q: 1-blue_probab_line(t,q) # I do not really use these, since they are just 1-blue_probab
# # # Flat form # # #
blue_probab_flat =   lambda t,q: q 
purple_probab_flat = lambda t,q: 1-blue_probab_flat(t,q) # I do not really use these, since they are just 1-blue_probab

if plot == True:
    # Visualize f(t) models given two plotting areas ax1 and ax2
    ax1, ax2, ax3 = plot_f_of_t(blue_probab_sine, purple_probab_sine,
                           blue_probab_line, purple_probab_line, 
                           blue_probab_flat, purple_probab_flat,
                           ax1, ax2, ax3, q = 0.2)
    fig_model.show()
```

## Division distribution¶

In [5]:

```
# Defining cell cycle lengths

# Distribution of Cell Cycle Lengths A (Purple)
mean_cell_cycle_A = 15 # hours
cell_cycle_dist_A =  7 
cell_cycle_dist_loc_A = 0.8 * mean_cell_cycle_A
cell_cycle_dist_scale_A = 0.05 * mean_cell_cycle_A


# Distribution of Cell Cycle Lengths P (Green)
mean_cell_cycle_P = 15
cell_cycle_dist_P =  7
cell_cycle_dist_loc_P = 0.8 * mean_cell_cycle_P
cell_cycle_dist_scale_P = 0.05 * mean_cell_cycle_P        


def draw_random_cell_cycle_length(celltype):
    if celltype == 'P':
        cycle = gamma.rvs(a     = cell_cycle_dist_A,
                          loc   = cell_cycle_dist_loc_A,
                          scale = cell_cycle_dist_scale_A)
    elif celltype == 'G':
        cycle = gamma.rvs(a     = cell_cycle_dist_P,
                          loc   = cell_cycle_dist_loc_P,
                          scale = cell_cycle_dist_scale_P)
    elif celltype == 'B':
        cycle = np.nan
        
    return cycle

if plot == True:
    # Plotting the division distributions given a drawing area ax3
    ax3 = plot_division_distributions(cell_cycle_dist_A, cell_cycle_dist_loc_A, cell_cycle_dist_scale_A, 
                                      cell_cycle_dist_P, cell_cycle_dist_loc_P, cell_cycle_dist_scale_P, 
                                      ax4)
    fig_model.show()
```

In [6]:

```
# # Plotting the division distributions given a drawing area ax3
# fig = plt.figure()
# ax4 = fig.add_subplot(111)
# ax3 = plot_division_distributions(cell_cycle_dist_A, cell_cycle_dist_loc_A, cell_cycle_dist_scale_A, 
#                                   cell_cycle_dist_P, cell_cycle_dist_loc_P, cell_cycle_dist_scale_P, 
#                                   ax4)
# fig.show()
# fig.savefig('cellcycledist.svg')
```

## State transitions (c,q,model)¶

In [7]:

```
def state_transitions(c,q,model):
    #States = ['B', 'G', 'P'] # BLUE GREEN PURPLE

    if model == 'sin':
        f = lambda t, q: blue_probab_sine(t,q)
    elif model == 'line':
        f = lambda t, q: blue_probab_line(t,q)

    BB = 1;                     BG = 0;       BP = 0
    GB = lambda t: c*f(t,q);    GG = 1-c;     GP = lambda t: c*(1-f(t,q))
    PB = 0;                     PG = 0;       PP = 1

    
    cumsum_list = lambda t: [GG, GG+GB(t),1]
    STdict = {'BB':BB,'BG':BG,'BP':BP,
              'GB':GB,'GG':GG,'GP':GP,
              'PB':PB,'PG':PG,'PP':PP,}
    
    return STdict
```

## Get offspring¶

In [8]:

```
def get_offspring(cell_type,STdict,time):    
    rand = random.random()
    # # # Celltype G # # #
    if cell_type == 'G':
        if rand < STdict['GG']:         # This it the chance that it will Be G-->G
            return 'G'
        elif rand < STdict['GG']+STdict['GB'](time): # If it did not go to G, but is still below GG+GB(t), then it must be in a range of size GB(t), so this is the G->B
            return 'B'
        else:                 # if it did not go to any of the others, then because to probabs sum to 1, it must be G->P
            return 'P'
    
    # # # Celltype P # # #   
    elif cell_type == 'P':
        if rand < STdict['PG']:         # This it the chance that it will Be P -->G
            return 'G'
        elif rand < STdict['PG']+STdict['PB']: # If it did not go to G, but is still below PG+PB, then it must be in a range of size PB, so this is the P->B
            return 'B'
        else:                 # if it did not go to any of the others, then because to probabs sum to 1, it must be P->P
            return 'P'
    
    # # # Celltype B # # #
    elif cell_type == 'B':
        if rand < STdict['BG']:         # This it the chance that it will Be B-->G
            return 'G'
        elif rand < STdict['BG']+STdict['BB']: # If it did not go to G, but is still below BG+BB, then it must be in a range of size BB, so this is the B->B
            return 'B'
        else:                 # if it did not go to any of the others, then because to probabs sum to 1, it must be B->P
            return 'P'
```

# Make the cells divide¶

## Simulation code¶

In [9]:

```
# Simulation code
def time_convert_228h_348h_to_0_1(input_in_hours): # 348 is 14.5, 228 is 9.5
    return (input_in_hours - 228)/(348-228)

def cell_sim_make_init_condition_v_0_1(c,q,model,n_max_cells):
    cd = {0: [0,dict()]} # CellDict
    n = n_max_cells
    
    init_cells = pd.DataFrame({ 'DICT'             :            [{} for i in range(n)],
                                'generation'       : np.array(  n * [0 ]),
                                'next_divide_time' : np.array(  n * [np.nan]),
                                'offspring'        :            n * [[] ],
                                'parent'           : np.array(  n * [np.nan]),
                                'time_born'        : np.array(  n * [0 ]),
                                'times_divided'    : np.array(  n * [0 ]),
                                'type'             : n * ['G'],
                              })
    #### List init cells here ####
    ndt = draw_random_cell_cycle_length
    ################  [ 'DICT'  , 'generation', 'next_divide_time'         , 'offspring', 'parent', 'time_born', 'times_divided', 'type']
    init_cells.loc[0]=[ cd[0][1],       0     ,  9.5*24 + random.random()*ndt('G'),      []    ,  np.nan ,    9.5*24  ,       0        ,   'G']
    #init_cells.loc[1]=[ cd[1][1],       0    ,         0                  ,      []    ,  np.nan ,       0    ,       0        ,   'G']
    #init_cells.loc[2]=[ cd[2][1],       0    ,         0                  ,      []    ,  np.nan ,       0    ,       0        ,   'G']
    
    return init_cells, cd

def cell_sim(init_cells, cd, c, q, model, n_max_cells,timestop, DictStuff = 0):
    STdict = state_transitions(c,q,model)
    cells = copy(init_cells)
    n_cells = 1
    time_divided = 0
    
    while time_divided < timestop * 24: # 14.5 * 24:
    #for i in range(n_max_cells):
        # Find cell to divide
        idx_to_divide = cells['next_divide_time'].idxmin(skipna = True)
        if np.isnan(idx_to_divide):
            break
            
        type_to_divide = cells['type'][idx_to_divide]
        time_divided = cells['next_divide_time'][idx_to_divide]
        
        parent_idx = idx_to_divide
        offspring_idx = n_cells  # Since python 0 indexes, the idx of the new cell should be same as n_current_cells

        time = time_convert_228h_348h_to_0_1(time_divided) #time_divided, scaled to a range between 0 and 1
        
        # Create offspring
        offspring_generation = cells['generation'][parent_idx] + 1
        offspring_type = get_offspring(type_to_divide,STdict,time)
        offspring_times_divided = 0 # Obviously
        offsprint_parent = parent_idx
        offspring_time_born = time_divided
        offspring_next_divide_time = offspring_time_born + draw_random_cell_cycle_length(offspring_type)

        cells.loc[offspring_idx]=[{},offspring_generation, offspring_next_divide_time, [], offsprint_parent, offspring_time_born, offspring_times_divided, offspring_type] 

        # Update parent cell
        parents_new_type = get_offspring(type_to_divide,STdict,time)
        cells.loc[parent_idx,'type'] = parents_new_type
        cells.loc[parent_idx,'next_divide_time'] = time_divided + draw_random_cell_cycle_length(parents_new_type)
        cells.loc[parent_idx,'times_divided'] += 1
        cells.loc[parent_idx,'offspring'].append(offspring_idx)

        if DictStuff == 1:
            # Make CellDict for heritage plotting
            cells.loc[parent_idx,'DICT'][parent_idx]    = [1, offspring_time_born, {}]
            cells.loc[parent_idx,'DICT'][offspring_idx] = [1, offspring_time_born, {}]
            cells.set_value(offspring_idx, 'DICT', cells.loc[parent_idx,'DICT'][offspring_idx][2]) 
            cells.set_value(parent_idx, 'DICT', cells.loc[parent_idx,'DICT'][parent_idx][2])
        
        n_cells += 1
    return cells, cd, n_cells

def run_cell_sim(c,q,model,n_max_cells,timestop, DictStuff = 0):
    init_cells, cd = cell_sim_make_init_condition_v_0_1(c,q,model,n_max_cells)
    simu_cells, cd, n_cells = cell_sim(init_cells,cd,c,q,model,n_max_cells,timestop, DictStuff = DictStuff)
    decendant_info = get_decendants_numbers(simu_cells)
    return simu_cells[0:n_cells], cd, decendant_info, n_cells
```

# Load Parameter scan saves¶

In [10]:

```
c_index = np.linspace(0,0.4,20)
q_index = np.linspace(0,1  ,20)
c_index = c_index[1:]
q_index = q_index[0:-1]

dfs_with_data = {}
dfs_with_times = {}

# Load information to load data and times
dfs_with_data['sine'] = glob.glob('paramscan_data_df_c0to04_sine_withfixedtimes_[0-9]*.json')
dfs_with_times['sine'] = glob.glob('paramscan_times_df_c0to04_sine_withfixedtimes_[0-9]*.json')
print(Back.WHITE + '### sine_withtimes_fixsinus ###'+ Fore.RESET)
pprint(dfs_with_data['sine'])
pprint(dfs_with_times['sine'])

# Load information to load data and times
dfs_with_data['flat'] = glob.glob('paramscan_data_df_c0to04_flat_withfixedtimes_[0-9]*.json')
dfs_with_times['flat'] = glob.glob('paramscan_times_df_c0to04_flat_withfixedtimes_[0-9]*.json')
print(Back.WHITE + '### flat_withtimes - NOT FIXED ###' + Fore.RESET)
pprint(dfs_with_data['flat'])
pprint(dfs_with_times['flat'])
```

```
### sine_withtimes_fixsinus ###
['paramscan_data_df_c0to04_sine_withfixedtimes_12.json',
 'paramscan_data_df_c0to04_sine_withfixedtimes_6.json',
 'paramscan_data_df_c0to04_sine_withfixedtimes_11.json',
 'paramscan_data_df_c0to04_sine_withfixedtimes_4.json',
 'paramscan_data_df_c0to04_sine_withfixedtimes_1.json',
 'paramscan_data_df_c0to04_sine_withfixedtimes_3.json',
 'paramscan_data_df_c0to04_sine_withfixedtimes_2.json',
 'paramscan_data_df_c0to04_sine_withfixedtimes_5.json']
['paramscan_times_df_c0to04_sine_withfixedtimes_2.json',
 'paramscan_times_df_c0to04_sine_withfixedtimes_4.json',
 'paramscan_times_df_c0to04_sine_withfixedtimes_3.json',
 'paramscan_times_df_c0to04_sine_withfixedtimes_12.json',
 'paramscan_times_df_c0to04_sine_withfixedtimes_11.json',
 'paramscan_times_df_c0to04_sine_withfixedtimes_6.json',
 'paramscan_times_df_c0to04_sine_withfixedtimes_1.json',
 'paramscan_times_df_c0to04_sine_withfixedtimes_5.json']
### flat_withtimes - NOT FIXED ###
['paramscan_data_df_c0to04_flat_withfixedtimes_6.json',
 'paramscan_data_df_c0to04_flat_withfixedtimes_2.json',
 'paramscan_data_df_c0to04_flat_withfixedtimes_4.json',
 'paramscan_data_df_c0to04_flat_withfixedtimes_16.json',
 'paramscan_data_df_c0to04_flat_withfixedtimes_5.json',
 'paramscan_data_df_c0to04_flat_withfixedtimes_3.json',
 'paramscan_data_df_c0to04_flat_withfixedtimes_1.json',
 'paramscan_data_df_c0to04_flat_withfixedtimes_15.json']
['paramscan_times_df_c0to04_flat_withfixedtimes_4.json',
 'paramscan_times_df_c0to04_flat_withfixedtimes_15.json',
 'paramscan_times_df_c0to04_flat_withfixedtimes_3.json',
 'paramscan_times_df_c0to04_flat_withfixedtimes_5.json',
 'paramscan_times_df_c0to04_flat_withfixedtimes_1.json',
 'paramscan_times_df_c0to04_flat_withfixedtimes_6.json',
 'paramscan_times_df_c0to04_flat_withfixedtimes_16.json',
 'paramscan_times_df_c0to04_flat_withfixedtimes_2.json']
```

In [11]:

```
# Load all the data dfs and combine them

def load_and_combine_data_df(dfs_with_data):
    paramscan_data_df  = deepcopy(pd.read_json(dfs_with_data[0]))
    # fix
    paramscan_data_df.index = q_index
    paramscan_data_df.columns = c_index

    keys = list(paramscan_data_df.iloc[0,0].keys())

    for i in range(1, len(dfs_with_data)):
        paramscan_data_df2  = deepcopy(pd.read_json(dfs_with_data[i]))
        # fix
        paramscan_data_df2.index = q_index
        paramscan_data_df2.columns = c_index
        for key in keys:
            for q_n in range(19):
                for c_n in range(19):
                    paramscan_data_df.iloc[q_n,c_n][key] = paramscan_data_df.iloc[q_n,c_n][key] + paramscan_data_df2.iloc[q_n,c_n][key]
    del paramscan_data_df2
    return paramscan_data_df

# Load all the times dfs and combine them
def load_and_combine_times_df(dfs_with_times):
    paramscan_times_df  = deepcopy(pd.read_json(dfs_with_times[0]))
    # fix
    paramscan_times_df.index = q_index
    paramscan_times_df.columns = c_index

    keys = list(paramscan_times_df.iloc[0,0].keys())

    for i in range(1, len(dfs_with_times)):
        paramscan_times_df2  = deepcopy(pd.read_json(dfs_with_times[i]))
        # fix
        paramscan_times_df2.index = q_index
        paramscan_times_df2.columns = c_index
        for key in keys:
            for q_n in range(19):
                for c_n in range(19):
                    paramscan_times_df.iloc[q_n,c_n][key] = paramscan_times_df.iloc[q_n,c_n][key] + paramscan_times_df2.iloc[q_n,c_n][key]
    del paramscan_times_df2  
    
    # Must convert freq into... not freq, but just numbers at time:
    paramscan_times_df2 = deepcopy(paramscan_times_df)
    keys = list(paramscan_times_df2.iloc[0,0].keys())
    for q_n in range(19):
        for c_n in range(19):
            for key in keys:
                if key == 'G_times':
                    paramscan_times_df2.iloc[q_n,c_n][key] = np.cumsum(paramscan_times_df2.iloc[q_n,c_n][key], axis = 1) + 1
                elif key == 'total_times':
                    paramscan_times_df2.iloc[q_n,c_n][key] = np.cumsum(paramscan_times_df2.iloc[q_n,c_n][key], axis = 1) + 1
                else:
                    paramscan_times_df2.iloc[q_n,c_n][key] = np.cumsum(paramscan_times_df2.iloc[q_n,c_n][key], axis = 1)
    return paramscan_times_df2
```

In [12]:

```
paramscan_data_df_dict = {}
paramscan_times_df2_dict = {}

for key in dfs_with_data.keys():
    paramscan_data_df_dict[key]   = deepcopy(load_and_combine_data_df( dfs_with_data[key]))
for key in dfs_with_times.keys():
    paramscan_times_df2_dict[key] = deepcopy(load_and_combine_times_df(dfs_with_times[key]))
```

In [13]:

```
np.shape(paramscan_times_df2_dict['sine'].iloc[0,0]['B_times'])
```

Out[13]:

```
(2400, 4)
```

## Visulizing paramscan\_data\_df¶

In [14]:

```
# Nice bar
def nice_bars(paramscan_data_df, n_q = 13, n_c = 5):
    #n_q = 0; 
    #n_c = 6;
    i_c = paramscan_data_df.columns.values[n_c]
    i_q = paramscan_data_df.index[n_q]

    Total = copy(paramscan_data_df.loc[i_q,i_c]['total']) 
    B     = copy(paramscan_data_df.loc[i_q,i_c]['B'])
    G     = copy(paramscan_data_df.loc[i_q,i_c]['G']) 
    P     = copy(paramscan_data_df.loc[i_q,i_c]['P'])
    b_for_all, g_for_all, p_for_all, totals, idx_1, idx_2, idx_3 = sort_and_group(Total, B, G, P)

    # # # ax1
    fig, ax1 = plot_sort_and_group(b_for_all, g_for_all, p_for_all, totals, idx_1, idx_2, idx_3, subplotlayout=211)
    ax1.set_title('Histogram')
    fig.show()

    # # # for ax2 and ax3
    try:    group_3_p_ratios       = p_for_all[idx_3] / totals[idx_3]
    except: group_3_p_ratios       = 10 * [0]
    # # ax2 
    ax2 = fig.add_subplot(223);
    ax2.hist(group_3_p_ratios, bins = 100, color = 'purple')
    ax2.set_title('Hist of purple ratio', loc='left', color = 'purple')
    ax2.set_xlabel('Variate (purple ratio in group 3)')
    ax2.tick_params('y', colors='purple')
    ax2.set_ylabel('Occurances', color = 'purple')
    # # ax3
    ecdf = ECDF(sorted(group_3_p_ratios))
    ax3 = ax2.twinx()
    y = sorted(group_3_p_ratios)
    ax3.plot(y, ecdf(y),'-',color = 'black')
    ax3.tick_params('y', colors='k')
    ax3.set_title('ECDF of purple ratio', loc='right')
    ax3.set_xlabel('Variate (purple ratio in group 3)')
    ax3.set_ylabel('Cumulative probab')

    fig.suptitle('q : ' + str(np.round(paramscan_data_df.index[n_q],2))+ '  and  c : ' +str(np.round(paramscan_data_df.columns.values[n_c],2) ) )
    fig.tight_layout()
    fig.show()

    # # # for ax4 and ax5
    try:    group_2_b_ratios       = b_for_all[idx_2] / totals[idx_2]
    except: group_2_b_ratios       = 10 * [0]
    # # ax2 
    ax4 = fig.add_subplot(224);
    ax4.hist(group_2_b_ratios, bins = 100, color = 'blue')
    ax4.set_title('Hist of blue ratio', loc='left', color = 'blue')
    ax4.set_xlabel('Variate (blue ratio in group 2)')
    ax4.tick_params('y', colors='blue')
    ax4.set_ylabel('Occurances', color = 'blue')
    # # ax3
    ecdf = ECDF(sorted(group_2_b_ratios))
    ax5 = ax4.twinx()
    y = sorted(group_3_p_ratios)
    ax5.plot(y, ecdf(y),'-',color = 'black')
    ax5.tick_params('y', colors='k')
    ax5.set_title('ECDF of blue ratio', loc='right')
    ax5.set_xlabel('Variate (blue ratio in group 2)')
    ax5.set_ylabel('Cumulative probab')

    fig.suptitle('q : ' + str(np.round(paramscan_data_df.index[n_q],2))+ '  and  c : ' +str(np.round(paramscan_data_df.columns.values[n_c],2) ) )
    fig.tight_layout()
    fig.show()
```

In [15]:

```
if plot == True:
    #nice_bars(paramscan_data_df_dict['sine_notfixed'], n_q = 5, n_c = 5)

    fx = 'sine'
    n_q = 10; 
    n_c = 8;
    i_c = paramscan_data_df_dict[fx].columns.values[n_c]
    i_q = paramscan_data_df_dict[fx].index[n_q]

    Total = np.array(copy(paramscan_data_df_dict[fx].loc[i_q,i_c]['total']) )
    B     = np.array(copy(paramscan_data_df_dict[fx].loc[i_q,i_c]['B']) )
    G     = np.array(copy(paramscan_data_df_dict[fx].loc[i_q,i_c]['G']) )
    P     = np.array(copy(paramscan_data_df_dict[fx].loc[i_q,i_c]['P']) )

    idx_to_plot = random.sample(range(len(Total)),50)


    b_for_all, g_for_all, p_for_all, totals, idx_1, idx_2, idx_3 = sort_and_group(Total[idx_to_plot], B[idx_to_plot], G[idx_to_plot], P[idx_to_plot])

    fig, ax1 = plot_sort_and_group(b_for_all, g_for_all, p_for_all, totals, idx_1, idx_2, idx_3, subplotlayout=111, plotboxes=False, boxoffset=-0.1)
    ax1.set_title('Histogram')
    fig.show()
```

In [16]:

```
fig.savefig('z_sinus_optiparam_test.svg')
```

In [ ]:

```

```

In [17]:

```
# Purple kde
def show_purple_kde(paramscan_data_df, n_q = 5, n_c = 5, bandwidth = 0.015):
    #n_q = 13; 
    #n_c = 5;
    i_c = paramscan_data_df.columns.values[n_c]
    i_q = paramscan_data_df.index[n_q]
    Total = copy(paramscan_data_df.loc[i_q,i_c]['total']) 
    B     = copy(paramscan_data_df.loc[i_q,i_c]['B'])
    G     = copy(paramscan_data_df.loc[i_q,i_c]['G']) 
    P     = copy(paramscan_data_df.loc[i_q,i_c]['P'])
    b_for_all, g_for_all, p_for_all, totals, idx_1, idx_2, idx_3 = sort_and_group(Total, B, G, P)
    try:    group_3_p_ratios       = p_for_all[idx_3] / totals[idx_3]
    except: group_3_p_ratios       = 10 * [0]
    ecdf = ECDF(sorted(group_3_p_ratios))

    # Make figure    
    fig = plt.figure()
    ax = fig.add_subplot(111)

    # Histogram
    ax.hist(group_3_p_ratios, normed=1, bins = 50, color = 'purple', alpha = 0.8)
    ax.set_ylabel('Normalized frequency', color='purple')
    ax.tick_params('y', colors='purple')
    ax.set_xlabel('ratio')

    # Red
    kde = KernelDensity(kernel='gaussian', bandwidth=bandwidth).fit(group_3_p_ratios[:, np.newaxis])
    X_plot = np.linspace(0, 1, 10000)[:, np.newaxis]
    y = kde.score_samples(X_plot)

    ax2 = ax.twinx()
    ax.plot(X_plot, np.exp(y), 'k', linewidth = 3)
    ax2.plot(X_plot, np.exp(y), 'white', linewidth = 3, alpha = 0)
    ax2.set_ylabel('KDE', color='k')
    ax2.tick_params('y', colors='k')
    plt.yticks(np.array([0,0.5,1,1.5,2,2.5,3])*1.05-0.2,[0,0.5,1,1.5,2,2.5,3])
    
    fig.tight_layout()

    #ax.plot(X_plot, max(pdf) * 1/len(X_plot)*np.cumsum(np.exp(y)), 'r')
    fig.show()
```

In [18]:

```
if plot == True:
    #nice_bars(paramscan_data_df_dict['sine_notfixed'], n_q = 5, n_c = 5)
    show_purple_kde(paramscan_data_df_dict['sine'], n_q = 4, n_c = 4, bandwidth = 0.015)
    #show_purple_kde(paramscan_data_df_dict['sine_notfixed'], n_q = 5, n_c = 5, bandwidth = 0.015)
    #fig = plt.gcf()
    #fig.savefig('purple_dist4-4.svg')
```

# Load Article 4d & 1e plots & S7d¶

In [19]:

```
fig4d = pd.read_excel('E95_to_145_tracing_quantification_2.xlsx',sheetname='Sorted',skip_footer=3)
fig1e = pd.read_excel('LineageTracing_Rosa26CreERmTmG_e14.5_Anne_Alex.xlsx',sheetname='This')
```

In [20]:

```
# Preparing data
fig4d1e_b = list(fig4d['Endocrine'])  + list(fig1e['Blue'])
fig4d1e_g = list(fig4d['Progenitors']) + list(fig1e['Green'])
fig4d1e_p = list(fig4d['Acinar']) + list(fig1e['Purple'])
fig4d1e_totals = list(fig4d['Total sum.']) + list(fig1e['Blue'] + fig1e['Purple'] + fig1e['Green'])

# sort and group
fig4d1e_b_for_all, fig4d1e_g_for_all, fig4d1e_p_for_all, fig4d1e_totals, fig4d1e_idx_1, fig4d1e_idx_2, fig4d1e_idx_3 = sort_and_group(fig4d1e_totals, fig4d1e_b, fig4d1e_g, fig4d1e_p)

# Plotting
fig, ax1 = plot_sort_and_group(fig4d1e_b_for_all, fig4d1e_g_for_all, fig4d1e_p_for_all, fig4d1e_totals, fig4d1e_idx_1, fig4d1e_idx_2, fig4d1e_idx_3)
ax1.set_title('4d + 1e')
fig.show()
```

In [21]:

```
fig4d1e_group_3_p_ratios = np.array(sorted(fig4d1e_p_for_all[fig4d1e_idx_3] / fig4d1e_totals[fig4d1e_idx_3] ))
fig4d1e_group_3_b_ratios = np.array(sorted(fig4d1e_b_for_all[fig4d1e_idx_3] / fig4d1e_totals[fig4d1e_idx_3] ))
fig4d1e_group_2_p_ratios = np.array(sorted( [fig4d1e_p_for_all[fig4d1e_idx_2_i] / fig4d1e_totals[fig4d1e_idx_2_i] for fig4d1e_idx_2_i in fig4d1e_idx_2 if fig4d1e_totals[fig4d1e_idx_2_i] >= 29] ))
fig4d1e_group_2_b_ratios = np.array(sorted( [fig4d1e_b_for_all[fig4d1e_idx_2_i] / fig4d1e_totals[fig4d1e_idx_2_i] for fig4d1e_idx_2_i in fig4d1e_idx_2 if fig4d1e_totals[fig4d1e_idx_2_i] >= 29] ))

# fig = plt.figure()
# plt.plot(fig4d1e_group_3_p_ratios,'.', color = 'purple')
# plt.plot(fig4d1e_group_2_b_ratios,'.', color = 'blue')
# fig.show()
```

In [22]:

```
figS7 = pd.read_excel('ICR_WT_quantification.xlsx')
figS7_ratios = figS7.loc[[0,1,4,5,8,9,12,13],['Sox9', 'Ptf1a', 'Ngn3','Sox9/All', 'Ptf1a/All', 'Ngn3/All', 'Unnamed: 11', 'Unnamed: 11', 'Unnamed: 11']].fillna(0)
figS7_ratios.index = ['14.5','14.5','12.5','12.5','11.5','11.5','10.5','10.5']
figS7_ratios.columns = ['G', 'P', 'B', 'G/T','P/T','B/T', 'G/T_sigma','P/T_sigma','B/T_sigma']
figS7_ratios

total = (figS7_ratios['G'] + figS7_ratios['B'] + figS7_ratios['P'])
figS7_ratios['G/T_sigma'] = np.sqrt((figS7_ratios['G']/total*(1-figS7_ratios['G']/total))/(total))
figS7_ratios['P/T_sigma'] = np.sqrt((figS7_ratios['P']/total*(1-figS7_ratios['P']/total))/(total))
figS7_ratios['B/T_sigma'] = np.sqrt((figS7_ratios['B']/total*(1-figS7_ratios['B']/total))/(total))
figS7_ratios
```

Out[22]:

|  | G | P | B | G/T | P/T | B/T | G/T\_sigma | P/T\_sigma | B/T\_sigma |
| --- | --- | --- | --- | --- | --- | --- | --- | --- | --- |
| 14.5 | 21456.0 | 13948.0 | 7079.0 | 0.505049 | 0.328320 | 0.166631 | 0.002426 | 0.002278 | 0.001808 |
| 14.5 | 27375.0 | 20249.0 | 9788.0 | 0.476817 | 0.352696 | 0.170487 | 0.002084 | 0.001994 | 0.001569 |
| 12.5 | 3958.0 | 954.0 | 64.0 | 0.795418 | 0.191720 | 0.012862 | 0.005719 | 0.005581 | 0.001597 |
| 12.5 | 4353.0 | 815.0 | 86.0 | 0.828512 | 0.155120 | 0.016368 | 0.005200 | 0.004994 | 0.001751 |
| 11.5 | 3210.0 | 726.0 | 99.0 | 0.795539 | 0.179926 | 0.024535 | 0.006349 | 0.006047 | 0.002435 |
| 11.5 | 2455.0 | 579.0 | 86.0 | 0.786859 | 0.185577 | 0.027564 | 0.007332 | 0.006960 | 0.002931 |
| 10.5 | 1202.0 | 0.0 | 107.0 | 0.918258 | 0.000000 | 0.081742 | 0.007572 | 0.000000 | 0.007572 |
| 10.5 | 1327.0 | 0.0 | 111.0 | 0.922809 | 0.000000 | 0.077191 | 0.007038 | 0.000000 | 0.007038 |

In [23]:

```
60/5001
```

Out[23]:

```
0.01199760047990402
```

# Probabs¶

## Group 3 - Purple ratios probabilities¶

In [24]:

```
def make_df_g3_p_ratios(paramscan_data_df):
    df_g3_p_ratios = deepcopy(paramscan_data_df)

    for i_c in c_index:
        for i_q in q_index:
            Total = copy(paramscan_data_df.loc[i_q,i_c]['total']) 
            B     = copy(paramscan_data_df.loc[i_q,i_c]['B'])
            G     = copy(paramscan_data_df.loc[i_q,i_c]['G']) 
            P     = copy(paramscan_data_df.loc[i_q,i_c]['P'])
            b_for_all, g_for_all, p_for_all, totals, idx_1, idx_2, idx_3 = sort_and_group(Total, B, G, P)
            try:    group_3_p_ratios       = p_for_all[idx_3] / totals[idx_3]
            except: group_3_p_ratios       = 10 * [0]

            if sum(group_3_p_ratios) != 0:
                kde = KernelDensity(kernel='gaussian', bandwidth=0.015).fit(group_3_p_ratios[:, np.newaxis])
                X_input = fig4d1e_group_3_p_ratios[:, np.newaxis]
                y = kde.score_samples(X_input)
                df_g3_p_ratios.loc[i_q, i_c] = sum(np.exp(y))
            else:
                df_g3_p_ratios.loc[i_q, i_c] = np.float64(-10)
    return df_g3_p_ratios


# Plotting
def plot_df_g3_p_ratios(df_g3_p_ratios, figwidth, figheight):
    df_g3_p_ratios = df_g3_p_ratios.apply(pd.to_numeric)

    fig    = plt.figure(figsize=[figwidth,figheight]);
    ax     = fig.add_subplot(111);
    pf = df_g3_p_ratios.as_matrix()
    pcolor = ax.pcolor(pf, cmap=plt.get_cmap('jet'));
    pcolor.set_clim(vmin=-10, vmax=45)
    fig.colorbar(pcolor);
    plt.xticks(np.arange(0.5, len(df_g3_p_ratios.columns), 1),   np.round(df_g3_p_ratios.columns,2));
    plt.yticks(np.arange(0.5, len(df_g3_p_ratios.index), 1), np.round(df_g3_p_ratios.index,2));
    plt.xlabel('c');
    plt.ylabel('q');
    plt.title('Relative probab of purple ratios of the empircal data coming from param set');
    fig.show()
```

In [25]:

```
# Make the likelihood matrices for all the datasets
df_g3_p_ratios_dict = {}

for key in dfs_with_data.keys():
    df_g3_p_ratios_dict[key]   = deepcopy(make_df_g3_p_ratios( paramscan_data_df_dict[key]))
```

In [26]:

```
plot_df_g3_p_ratios(df_g3_p_ratios_dict['sine'], figwidth = 4, figheight = 2)
plot_df_g3_p_ratios(df_g3_p_ratios_dict['flat'], figwidth = 4, figheight = 2)
# plot_df_g3_p_ratios(df_g3_p_ratios_dict['flat'], figwidth = 4, figheight = 2)
```

## Group 2 - Blue ratio probabilities¶

Seems to have very low sample sizes for some params... so it might not be too good a meassure. For low Q there simply isnt any group 2.. all samples have purple in them.

In [ ]:

```

```

In [ ]:

```

```

In [27]:

```
def make_df_g2_b_ratios(paramscan_data_df):
    df_g2_b_ratios = deepcopy(paramscan_data_df)

    for i_c in c_index:
        for i_q in q_index:
            Total = copy(df_g2_b_ratios.loc[i_q,i_c]['total']) 
            B     = copy(df_g2_b_ratios.loc[i_q,i_c]['B'])
            G     = copy(df_g2_b_ratios.loc[i_q,i_c]['G']) 
            P     = copy(df_g2_b_ratios.loc[i_q,i_c]['P'])
            b_for_all, g_for_all, p_for_all, totals, idx_1, idx_2, idx_3 = sort_and_group(Total, B, G, P) 
            try:
                if len(idx_2) == 0:
                    group_2_b_ratios       = np.array(list(b_for_all[idx_3])) / np.array(list(totals[idx_3]))
                else:
                    group_2_b_ratios       = np.array(list(b_for_all[idx_2])+list(b_for_all[idx_3])) / np.array(list(totals[idx_2])+list(totals[idx_3]))
            except: 
                print(i_q, i_c)
                group_2_b_ratios       = 10 * [0]

            if sum(group_2_b_ratios) != 0:
                kde = KernelDensity(kernel='gaussian', bandwidth=0.005).fit(group_2_b_ratios[:, np.newaxis])
                X_input = np.array(list(fig4d1e_group_2_b_ratios[:])+list(fig4d1e_group_3_b_ratios[:]))[:,np.newaxis]
                y = kde.score_samples(X_input)
                df_g2_b_ratios.loc[i_q, i_c] = sum(np.exp(y))
            else:
                df_g2_b_ratios.loc[i_q, i_c] = np.float64(-10)
    df_g2_b_ratios = df_g2_b_ratios.apply(pd.to_numeric)
    return df_g2_b_ratios

def make_df_g2_b_ratios2(paramscan_data_df):
    df_g2_b_ratios = deepcopy(paramscan_data_df)

    for i_c in c_index:
        for i_q in q_index:
            Total = copy(paramscan_data_df.loc[i_q,i_c]['total']) 
            B     = copy(paramscan_data_df.loc[i_q,i_c]['B'])
            G     = copy(paramscan_data_df.loc[i_q,i_c]['G']) 
            P     = copy(paramscan_data_df.loc[i_q,i_c]['P'])
            b_for_all, g_for_all, p_for_all, totals, idx_1, idx_2, idx_3 = sort_and_group(Total, B, G, P)
            try:    group_2_b_ratios       = b_for_all[idx_2] / totals[idx_2]
            except: group_2_b_ratios       = 10 * [0]

            if sum(group_2_b_ratios) != 0:
                kde = KernelDensity(kernel='gaussian', bandwidth=0.005).fit(group_2_b_ratios[:, np.newaxis])
                X_input = fig4d1e_group_2_b_ratios[:, np.newaxis]
                y = kde.score_samples(X_input)
                df_g2_b_ratios.loc[i_q, i_c] = sum(np.exp(y))
            else:
                df_g2_b_ratios.loc[i_q, i_c] = np.float64(-10)
    df_g2_b_ratios = df_g2_b_ratios.apply(pd.to_numeric)
    return df_g2_b_ratios

def plot_df_g2_b_ratios(df_g2_b_ratios, figwidth, figheight):
    # Plotting
    fig    = plt.figure(figsize=[figwidth,figheight]);
    ax     = fig.add_subplot(111);
    pcolor = ax.pcolor(df_g2_b_ratios.apply(pd.to_numeric), cmap=plt.get_cmap('jet'));
    fig.colorbar(pcolor);
    plt.xticks(np.arange(0.5, len(df_g2_b_ratios.columns), 1),   np.round(df_g2_b_ratios.columns,2));
    plt.yticks(np.arange(0.5, len(df_g2_b_ratios.index), 1), np.round(df_g2_b_ratios.index,2));
    plt.xlabel('c');
    plt.ylabel('q');
    plt.title('Relative probab of blue ratios of the empircal data coming from param set');

    # # Hover tooltip
    # indices = []
    # for i in range(20):
    #     for j in range(20):
    #         indices.append((i,j))

    # labels = ['median is '+ str(np.round(elem,2)) + str(indices[n]) for n, elem in enumerate(df.values.flatten())]
    # tooltip = mpld3.plugins.PointLabelTooltip(pcolor, labels=labels)
    # mpld3.plugins.connect(fig, tooltip)
    # mpld3.display()
    fig.show()
```

In [28]:

```
# Make the likelihood matrices for all the datasets
df_g2_b_ratios_dict = {}

for key in dfs_with_data.keys():
    df_g2_b_ratios_dict[key]   = deepcopy(make_df_g2_b_ratios( paramscan_data_df_dict[key]))
```

In [29]:

```
plot_df_g2_b_ratios(df_g2_b_ratios_dict['sine'], figwidth = 4, figheight = 2)
plot_df_g2_b_ratios(df_g2_b_ratios_dict['flat'], figwidth = 4, figheight = 2)
# plot_df_g2_b_ratios(df_g2_b_ratios_dict['flat'], figwidth = 4, figheight = 2)
```

## Freq ratios - At time 10.5, 11.5, 12.5, 14.5¶

In [30]:

```
def ratios_at_timepoints(q_i, c_i, times_df):
    df = times_df
    b_ratios_at_timepoint = {} 
    g_ratios_at_timepoint = {}
    p_ratios_at_timepoint = {}
    for timepoint_n in range(4): # timepoint 0 is 9.5-(10.5). 1 is 10.5-(11.5). 2 is 11.5-(12.5). 3 is 12.5-(14.5)
        idx = np.array(df.loc[q_i,c_i]['total_times'])[:,timepoint_n] != 0 # only make ratios where possible, ie where there is a frequency above total of 0
        b_ratios_at_timepoint[timepoint_n] = np.array(df.loc[q_i,c_i]['B_times'])[:,timepoint_n][idx] / np.array(df.loc[q_i,c_i]['total_times'])[:,timepoint_n][idx]
        g_ratios_at_timepoint[timepoint_n] = np.array(df.loc[q_i,c_i]['G_times'])[:,timepoint_n][idx] / np.array(df.loc[q_i,c_i]['total_times'])[:,timepoint_n][idx]
        p_ratios_at_timepoint[timepoint_n] = np.array(df.loc[q_i,c_i]['P_times'])[:,timepoint_n][idx] / np.array(df.loc[q_i,c_i]['total_times'])[:,timepoint_n][idx]

    # THESE ARE NOT USED ANYWHERE    
    B_freq = np.array([np.mean(b_ratios_at_timepoint[timepoint_n]) for timepoint_n in range(4)])
    G_freq = np.array([np.mean(g_ratios_at_timepoint[timepoint_n]) for timepoint_n in range(4)])
    P_freq = np.array([np.mean(p_ratios_at_timepoint[timepoint_n]) for timepoint_n in range(4)])

    # These are used.
    B_freq_std = np.array([np.std(b_ratios_at_timepoint[timepoint_n]) for timepoint_n in range(4)])
    G_freq_std = np.array([np.std(g_ratios_at_timepoint[timepoint_n]) for timepoint_n in range(4)])
    P_freq_std = np.array([np.std(p_ratios_at_timepoint[timepoint_n]) for timepoint_n in range(4)])
    
    return b_ratios_at_timepoint, g_ratios_at_timepoint, p_ratios_at_timepoint, B_freq, G_freq, P_freq

def ratios_at_timepoints2(q_i, c_i, times_df):
    df = times_df
    b_ratios_at_timepoint = {} 
    g_ratios_at_timepoint = {}
    p_ratios_at_timepoint = {}
    for timepoint_n in range(4): # timepoint 0 is 9.5-(10.5). 1 is 10.5-(11.5). 2 is 11.5-(12.5). 3 is 12.5-(14.5)
        idx = np.array(df.loc[q_i,c_i]['total_times'])[:,timepoint_n] != 0 # only make ratios where possible, ie where there is a frequency above total of 0
        b_ratios_at_timepoint[timepoint_n] = np.array(df.loc[q_i,c_i]['B_times'])[:,timepoint_n][idx] / np.array(df.loc[q_i,c_i]['total_times'])[:,timepoint_n][idx]
        g_ratios_at_timepoint[timepoint_n] = np.array(df.loc[q_i,c_i]['G_times'])[:,timepoint_n][idx] / np.array(df.loc[q_i,c_i]['total_times'])[:,timepoint_n][idx]
        p_ratios_at_timepoint[timepoint_n] = np.array(df.loc[q_i,c_i]['P_times'])[:,timepoint_n][idx] / np.array(df.loc[q_i,c_i]['total_times'])[:,timepoint_n][idx]

    sum_of_B = sum(np.array(df.loc[q_i,c_i]['B_times'])[:,:])
    sum_of_G = sum(np.array(df.loc[q_i,c_i]['G_times'])[:,:])
    sum_of_P = sum(np.array(df.loc[q_i,c_i]['P_times'])[:,:])
    sum_of_total = sum(np.array(df.loc[q_i,c_i]['total_times'])[:,:])
    B_freq = sum_of_B / sum_of_total
    G_freq = sum_of_G / sum_of_total
    P_freq = sum_of_P / sum_of_total
    B_freq_std = np.sqrt((B_freq*(1-B_freq))/ sum_of_total)
    G_freq_std = np.sqrt((G_freq*(1-G_freq))/ sum_of_total)
    P_freq_std = np.sqrt((P_freq*(1-P_freq))/ sum_of_total)    

    return b_ratios_at_timepoint, g_ratios_at_timepoint, p_ratios_at_timepoint, B_freq, G_freq, P_freq

def probabs_from_2d_kernel(figS7_ratios, kernel_105, kernel_115, kernel_125, kernel_145):
    figS7_ratios
    p_105_0 = kernel_105([np.array(figS7_ratios.loc['10.5','B/T'])[0], np.array(figS7_ratios.loc['10.5','P/T'])[0]])
    p_115_0 = kernel_115([np.array(figS7_ratios.loc['11.5','B/T'])[0], np.array(figS7_ratios.loc['11.5','P/T'])[0]])
    p_125_0 = kernel_125([np.array(figS7_ratios.loc['12.5','B/T'])[0], np.array(figS7_ratios.loc['12.5','P/T'])[0]])
    p_145_0 = kernel_145([np.array(figS7_ratios.loc['14.5','B/T'])[0], np.array(figS7_ratios.loc['14.5','P/T'])[0]])
    
    p_105_1 = kernel_105([np.array(figS7_ratios.loc['10.5','B/T'])[1], np.array(figS7_ratios.loc['10.5','P/T'])[1]])
    p_115_1 = kernel_115([np.array(figS7_ratios.loc['11.5','B/T'])[1], np.array(figS7_ratios.loc['11.5','P/T'])[1]])
    p_125_1 = kernel_125([np.array(figS7_ratios.loc['12.5','B/T'])[1], np.array(figS7_ratios.loc['12.5','P/T'])[1]])
    p_145_1 = kernel_145([np.array(figS7_ratios.loc['14.5','B/T'])[1], np.array(figS7_ratios.loc['14.5','P/T'])[1]])
    return p_105_0*p_105_1, p_115_0*p_115_1, p_125_0*p_125_1, p_145_0*p_145_1
    
def get_2dkernels_for_params(q_i, c_i, times_df, bw_method = 0.50):
    b_ratios_at_timepoint, g_ratios_at_timepoint, p_ratios_at_timepoint, B_freq, G_freq, P_freq = ratios_at_timepoints(q_i, c_i, times_df)
    b_105 = b_ratios_at_timepoint[0]
    p_105 = p_ratios_at_timepoint[0]
    values_105 = np.vstack([b_105, p_105])
    kernel_105 = stats.gaussian_kde(values_105, bw_method= bw_method)
    b_115 = b_ratios_at_timepoint[1]
    p_115 = p_ratios_at_timepoint[1]
    values_115 = np.vstack([b_115, p_115])
    kernel_115 = stats.gaussian_kde(values_115, bw_method= bw_method)
    b_125 = b_ratios_at_timepoint[2]
    p_125 = p_ratios_at_timepoint[2]
    values_125 = np.vstack([b_125, p_125])
    kernel_125 = stats.gaussian_kde(values_125, bw_method= bw_method)
    b_145 = b_ratios_at_timepoint[3]
    p_145 = p_ratios_at_timepoint[3]
    values_145 = np.vstack([b_145, p_145])
    kernel_145 = stats.gaussian_kde(values_145, bw_method= bw_method)
    return kernel_105, kernel_115, kernel_125, kernel_145

def get_2dkernels_for_params_minipancreas_with_ratios(q_i, c_i, times_df, bw_method = 0.50, bundle_size = 100, bundle_type = 'bootstrap_norefill', n_bootstraps_to_create = 1000):
    window_size = bundle_size
    window_type = bundle_type
    
    def give_ratios_for_window(window, times_df, q_i, c_i): # bad coding style to define func inside func.. but oh well,
        b_num_in_one_minipancreas = (   sum(np.array(times_df.loc[q_i,c_i]['B_times'])[window,:])   )
        p_num_in_one_minipancreas = (   sum(np.array(times_df.loc[q_i,c_i]['P_times'])[window,:])   )
        g_num_in_one_minipancreas = (   sum(np.array(times_df.loc[q_i,c_i]['G_times'])[window,:])   )
        total_num_in_one_minipancreas = (   sum(np.array(times_df.loc[q_i,c_i]['total_times'])[window,:])   )
        b_ratios_in_one_minipancreas = b_num_in_one_minipancreas / total_num_in_one_minipancreas
        p_ratios_in_one_minipancreas = p_num_in_one_minipancreas / total_num_in_one_minipancreas
        g_ratios_in_one_minipancreas = g_num_in_one_minipancreas / total_num_in_one_minipancreas
        return b_ratios_in_one_minipancreas, p_ratios_in_one_minipancreas, g_ratios_in_one_minipancreas

    if window_type == 'conservative':
        windows_list = []
        for i in range(int(np.floor(len(times_df.iloc[0,0]['B_times'])/window_size))):
            windows_list.append(range(0+i*window_size,window_size+i*window_size,1))
    elif window_type == 'bootstrap_refill':
        windows_list = []
        for i in range(n_bootstraps_to_create):
            windows_list.append(random.sample(range(len(times_df.iloc[0,0]['B_times'])),window_size)) 
    elif window_type == 'bootstrap_norefill':
        windows_list = []
        for i in range(n_bootstraps_to_create):
            windows_list.append(list(np.random.randint(0,len(times_df.iloc[0,0]['B_times']),window_size)))
    else:
        print(window_type)
    
    b_ratios_in_all_minipancreas = []
    p_ratios_in_all_minipancreas = []
    g_ratios_in_all_minipancreas = []
    for window in windows_list:
        b_ratios_in_on_minipancreas, p_ratios_in_on_minipancreas, g_ratios_in_on_minipancreas = give_ratios_for_window(window, times_df, q_i, c_i)
        b_ratios_in_all_minipancreas.append(b_ratios_in_on_minipancreas)
        p_ratios_in_all_minipancreas.append(p_ratios_in_on_minipancreas)
        g_ratios_in_all_minipancreas.append(g_ratios_in_on_minipancreas)
    
    b_ratios_in_all_minipancreas_np = np.array(b_ratios_in_all_minipancreas)[:,:]
    p_ratios_in_all_minipancreas_np = np.array(p_ratios_in_all_minipancreas)[:,:]
    g_ratios_in_all_minipancreas_np = np.array(g_ratios_in_all_minipancreas)[:,:]
    
    b_105 = b_ratios_in_all_minipancreas_np[:,0]
    p_105 = p_ratios_in_all_minipancreas_np[:,0]
    values_105 = np.vstack([b_105, p_105])
    kernel_105 = stats.gaussian_kde(values_105, bw_method = bw_method)
    b_115 = b_ratios_in_all_minipancreas_np[:,1]
    p_115 = p_ratios_in_all_minipancreas_np[:,1]
    values_115 = np.vstack([b_115, p_115])
    kernel_115 = stats.gaussian_kde(values_115, bw_method = bw_method)
    b_125 = b_ratios_in_all_minipancreas_np[:,2]
    p_125 = p_ratios_in_all_minipancreas_np[:,2]
    values_125 = np.vstack([b_125, p_125])
    kernel_125 = stats.gaussian_kde(values_125, bw_method = bw_method)
    b_145 = b_ratios_in_all_minipancreas_np[:,3]
    p_145 = p_ratios_in_all_minipancreas_np[:,3]
    values_145 = np.vstack([b_145, p_145])
    kernel_145 = stats.gaussian_kde(values_145, bw_method = bw_method)
    return kernel_105, kernel_115, kernel_125, kernel_145, b_ratios_in_all_minipancreas_np, p_ratios_in_all_minipancreas_np, g_ratios_in_all_minipancreas_np

def get_2dkernels_for_params_minipancreas(q_i, c_i, times_df, bw_method = 0.50, bundle_size = 100, bundle_type = 'bootstrap_norefill', n_bootstraps_to_create = 1000):
    kernel_105, kernel_115, kernel_125, kernel_145, b_ratios_in_all_minipancreas_np, p_ratios_in_all_minipancreas_np, g_ratios_in_all_minipancreas_np =  get_2dkernels_for_params_minipancreas_with_ratios(q_i, c_i, times_df, bw_method = bw_method, bundle_size = bundle_size, bundle_type = bundle_type, n_bootstraps_to_create = n_bootstraps_to_create)
    return kernel_105, kernel_115, kernel_125, kernel_145
```

In [31]:

```
# Test of probab from kernels
def test_kernel_2D_probab(paramscan_times_df2, q_n=4,c_n=4):
    #q_n = 4; c_n = 4
    q_i = q_index[q_n]
    c_i = c_index[c_n]
    try:
        del kernel_105, kernel_115, kernel_125, kernel_145
        del p_105, p_115, p_125, p_145
    except:
        pass
    kernel_105, kernel_115, kernel_125, kernel_145 = get_2dkernels_for_params_minipancreas(q_i, c_i, paramscan_times_df2, 
                                                                                           bundle_type = 'conservative', 
                                                                                           n_bootstraps_to_create = 12, bw_method = 2.00)
    p_105, p_115, p_125, p_145 = probabs_from_2d_kernel(figS7_ratios, kernel_105, kernel_115, kernel_125, kernel_145)
    #print(p_105, p_115, p_125, p_145)
    p_all = p_105 * p_115 * p_125 * p_145
    print(p_all)
    return [p_105 , p_115 , p_125 , p_145]
        
test_kernel_2D_probab(paramscan_times_df2_dict['sine'], q_n = 2, c_n = 4)
test_kernel_2D_probab(paramscan_times_df2_dict['flat'], q_n = 2, c_n = 4)
```

```
[  3.43537828e-71]
[ 0.]
```

Out[31]:

```
[array([  8.67953596e-22]),
 array([ 29677.05238209]),
 array([ 44.00783063]),
 array([ 0.])]
```

In [32]:

```
# def hahah()
#     fdgd
    
# a = 5
#     a = b
```

In [33]:

```
# probab from 2D kernels for all
def make_df_times_ratios(paramscan_times_df2, bw_method = 0.5, bundle_type = 'conservative', n_bootstraps_to_create = 1000):
    df_times_ratios = pd.DataFrame(np.zeros([19,19]))
    df_times_ratios.index = paramscan_times_df2.index
    df_times_ratios.columns = paramscan_times_df2.columns

    for q_n in range(19):
        for c_n in range(19):
            q_i = q_index[q_n]
            c_i = c_index[c_n]
            try:
                del kernel_105, kernel_115, kernel_125, kernel_145
                del p_105, p_115, p_125, p_145
            except:
                pass
            try:
                kernel_105, kernel_115, kernel_125, kernel_145 = get_2dkernels_for_params_minipancreas(q_i, c_i, paramscan_times_df2, 
                                                                                           bundle_type = bundle_type, 
                                                                                           n_bootstraps_to_create = n_bootstraps_to_create, bw_method = bw_method)
                p_105, p_115, p_125, p_145 = probabs_from_2d_kernel(figS7_ratios, kernel_105, kernel_115, kernel_125, kernel_145)
                #print(p_105, p_115, p_125, p_145)
                p_all = p_105 * p_115 * p_125 * p_145
            except:
                p_all = [-10]
            df_times_ratios.loc[q_i, c_i] = p_all[0]
        #print(q_n, 'done')
    return df_times_ratios
```

In [34]:

```
# Make the likelihood matrices for all the datasets
import pickle

bw_scan2 = pickle.load( open( "bw_scan2.p", "rb" ) )
bw_scan3 = pickle.load( open( "bw_scan3.p", "rb" ) )
bw_scan4 = pickle.load( open( "bw_scan4.p", "rb" ) )
    
bw_scan = {}
for key in bw_scan2.keys():
    bw_scan[key] = bw_scan2[key]
for key in bw_scan3.keys():
    bw_scan[key] = bw_scan3[key]
for key in bw_scan4.keys():
    bw_scan[key] = bw_scan4[key]
    
# bw_scan = {}
# for bw_method in np.arange(0.5,7.5,0.1):
#     Ds = []; keys = [];  AICs = [];  P = 0;  df = {};  df_log = {};  minlogs = [];  qs = {};  cs = {}; df_times = {}; df_times_log = {}     
#     for key in dfs_with_data.keys():
#         df_times[key]   = deepcopy(make_df_times_ratios( paramscan_times_df2_dict[key] , 
#                                                                    bw_method = bw_method,
#                                                                    bundle_type = 'conservative', n_bootstraps_to_create = 25))
#         df_times[key] = deepcopy(df_times[key] +10*(df_times[key] == -10))
#         df_times[key] = df_times[key].apply(pd.to_numeric)
#         df_times_log[key] = -2*np.log(df_times[key])
#         minlog  =df_times_log[key].stack().nsmallest(1)
#         q = list(minlog.index.values)[0][0]
#         c = list(minlog.index.values)[0][1]
        
#         Ds.append(np.max((df_times[key].max())))
        
#         AICs.append(2*2 -2*np.log(Ds[-1]))
#         minlogs.append(minlog)
#         qs[key] = q
#         cs[key] = c
#         keys.append(key)
        
#     P = np.exp((AICs[0]-AICs[1])/2)
#     print(bw_method)
#     bw_scan[bw_method] = [Ds, AICs, P, deepcopy(df_times), deepcopy(df_times_log), qs, cs, keys, minlogs]

# import pickle
# pickle.dump( bw_scan, open( "bw_scan.p", "wb" ) )
```

In [35]:

```
fig = plt.figure(figsize=[9,4])
ms = 1

dvalue = {'sine':df_g3_p_ratios_dict['sine'].iloc[7,6],
          'flat':df_g3_p_ratios_dict['flat'].iloc[16,6]}

ax1 = fig.add_subplot(131)
ax1.plot([key for key in bw_scan.keys()],([bw_scan[key][0][0]*dvalue['sine'] for key in bw_scan.keys()]),'ro', markersize = ms)
ax1.plot([key for key in bw_scan.keys()],([bw_scan[key][0][1]*dvalue['flat'] for key in bw_scan.keys()]),'bo', markersize = ms)
ax1.set_title('Likelihood')
# ax2 = fig.add_subplot(142)
# ax2.plot([key for key in bw_scan.keys()],(-2 * np.log([bw_scan[key][0][0] for key in bw_scan.keys()])),'ro')
# ax2.plot([key for key in bw_scan.keys()],(-2 * np.log([bw_scan[key][0][1] for key in bw_scan.keys()])),'bo')
# ax2.set_title('LogL')
ax3 = fig.add_subplot(132)
#ax3.plot([key for key in bw_scan.keys()],(([bw_scan[key][1][0] for key in bw_scan.keys()])),'ro', markersize = ms)
#ax3.plot([key for key in bw_scan.keys()],(([bw_scan[key][1][1] for key in bw_scan.keys()])),'bo', markersize = ms)
ax3.plot([key for key in bw_scan.keys()],(4 -2 * np.log([bw_scan[key][0][0]*dvalue['sine'] for key in bw_scan.keys()])),'ro', markersize = ms)
ax3.plot([key for key in bw_scan.keys()],(4 -2 * np.log([bw_scan[key][0][1]*dvalue['flat'] for key in bw_scan.keys()])),'bo', markersize = ms)
ax3.set_title('AIC')
ax4 = fig.add_subplot(133)
#ax4.plot(([key for key in bw_scan.keys()]),(([bw_scan[key][2] for key in bw_scan.keys()])),'ko', markersize = ms)
ys = np.exp((4 -2 * np.log([bw_scan[key][0][0]*dvalue['sine'] for key in bw_scan.keys()]) - (4 -2 * np.log([bw_scan[key][0][1]*dvalue['flat'] for key in bw_scan.keys()])))/2)
ax4.plot(([key for key in bw_scan.keys()]),ys,'ko', markersize = ms)

ax4.set_title('P')

xlim = [3,16]
ax1.set_xlim(xlim)
ax2.set_xlim(xlim)
ax3.set_xlim(xlim)
ax4.set_xlim(xlim)

# ax1.set_ylim([0,0.5])
ax2.set_ylim([-50,0])
ax3.set_ylim([-50,0])
ax4.set_ylim([0.,0.2])

ax1.set_xlabel('Bandwidth')
ax3.set_xlabel('Bandwidth')
ax4.set_xlabel('Bandwidth')

fig.savefig('z_bwscan.svg')
```

In [36]:

```
ys
```

Out[36]:

```
array([  1.91148452e+45,   6.72618482e+30,   1.29685653e+22,
         2.75815691e+16,   3.55948293e+12,   6.13343272e+09,
         5.85574044e+07,   1.78143458e+06,   1.20216425e+05,
         1.41570154e+04,   2.48287351e+03,   5.84407495e+02,
         1.72410515e+02,   6.08969743e+01,   2.49357598e+01,
         1.15539936e+01,   5.94156910e+00,   3.33648067e+00,
         2.01793919e+00,   1.29919428e+00,   8.81669852e-01,
         6.25516049e-01,   4.60815192e-01,   3.50550174e-01,
         2.74107119e-01,   2.19482665e-01,   1.79405538e-01,
         1.49313822e-01,   1.15737384e-01,   9.09946010e-02,
         7.29906836e-02,   5.96018953e-02,   4.94492582e-02,
         4.16145734e-02,   3.54723799e-02,   3.05877270e-02,
         2.66523961e-02,   2.41937495e-02,   2.66514053e-02,
         2.91857441e-02,   3.17872943e-02,   3.44467711e-02,
         3.71551726e-02,   3.95915935e-02,   4.11188546e-02,
         4.15271455e-02,   4.18523950e-02,   4.21617768e-02,
         4.24562996e-02,   4.27368898e-02,   4.30044001e-02,
         4.32596170e-02,   4.35032678e-02,   4.37360260e-02,
         4.39585165e-02,   4.41713198e-02,   4.43749760e-02,
         4.45699884e-02,   4.47568264e-02,   4.49359284e-02,
         4.51077041e-02,   4.52725370e-02,   4.54307862e-02,
         4.55827883e-02,   4.57288591e-02,   4.58692952e-02,
         4.60043752e-02,   4.61343610e-02,   4.62594992e-02,
         4.63800219e-02,   4.64961479e-02,   4.66080832e-02,
         4.67160225e-02,   4.68201494e-02,   4.69206373e-02,
         4.70176500e-02,   4.71113426e-02,   4.72018617e-02,
         4.72893460e-02,   4.73739270e-02,   4.74557292e-02,
         4.75348707e-02,   4.76114636e-02,   4.76856140e-02,
         4.47752530e-02,   4.17446320e-02,   3.90071335e-02,
         3.65280748e-02,   3.42775341e-02,   3.31706762e-02,
         3.37160837e-02,   3.42532928e-02,   3.47823377e-02,
         3.53032614e-02,   3.58161152e-02,   3.63209576e-02,
         3.68178537e-02,   3.73068746e-02,   3.77880965e-02,
         3.82616001e-02,   3.87274705e-02,   3.91857962e-02,
         3.96366688e-02,   4.00801828e-02,   4.05164348e-02,
         4.09455233e-02,   4.13675487e-02,   4.17826123e-02,
         4.21908166e-02,   4.25922649e-02,   4.29870610e-02,
         4.33753089e-02,   4.37571128e-02,   4.41325768e-02,
         4.45018048e-02,   4.48649002e-02,   4.52219661e-02,
         4.55731048e-02,   4.59184180e-02,   4.62580064e-02,
         4.65919699e-02,   4.65919699e-02,   4.69204075e-02,
         4.72434170e-02,   4.75610951e-02,   4.83430619e-02,
         5.01320890e-02,   5.19445359e-02,   5.37795381e-02,
         5.56362317e-02,   5.84932241e-02,   6.22963552e-02,
         6.62555452e-02,   7.03722754e-02,   7.46478590e-02,
         7.90834417e-02,   8.36800030e-02,   8.84383571e-02,
         9.33591551e-02,   9.84428874e-02,   1.03689886e-01,
         1.09100327e-01,   1.14674235e-01,   1.20411484e-01,
         1.26311806e-01,   1.32374789e-01,   1.38599884e-01,
         1.44986411e-01,   1.51533560e-01,   1.58240395e-01,
         1.65105865e-01,   1.72128799e-01,   1.79307921e-01,
         1.86641844e-01,   1.94129085e-01,   2.01768063e-01,
         2.09557105e-01,   2.17494453e-01,   2.25578268e-01,
         2.33806631e-01,   2.42177553e-01,   2.50688976e-01,
         2.59338780e-01,   2.68124783e-01,   2.77044752e-01,
         2.86096401e-01,   2.95277396e-01,   3.04585365e-01,
         3.14017895e-01,   3.23572536e-01,   3.33246812e-01,
         3.43038214e-01,   3.52944214e-01,   3.62962260e-01,
         3.73089783e-01,   3.83324201e-01,   3.93662919e-01,
         4.04103334e-01,   4.14642838e-01,   4.25278820e-01,
         4.24384992e-01,   4.20082993e-01,   4.15892470e-01,
         4.11809523e-01,   4.07830424e-01,   4.03951606e-01,
         4.00169652e-01,   3.96481294e-01,   3.92883398e-01,
         3.89372964e-01,   3.85947114e-01,   3.82603089e-01,
         3.79338242e-01,   3.76150035e-01,   3.73036029e-01,
         3.69993884e-01,   3.67021351e-01,   3.64116269e-01,
         3.67021351e-01,   3.40688043e-01,   3.19387385e-01,
         3.05282355e-01,   3.07689150e-01,   3.09828856e-01,
         3.11739073e-01,   3.13451095e-01,   3.14991119e-01,
         3.16381201e-01,   3.17640005e-01,   3.50989215e-01,
         4.37190225e-01,   5.33972821e-01,   5.11426144e-01,
         4.20261791e-01,   3.50958668e-01,   2.97334765e-01,
         2.55176257e-01,   2.21550578e-01,   1.94377781e-01,
         1.72156848e-01,   1.53787180e-01,   1.38449884e-01,
         1.25527389e-01,   1.14548107e-01,   1.20764246e-01,
         1.28769172e-01,   1.36761064e-01,   1.44720212e-01])
```

# Combine the two meassure and remove 0 probability places¶

### Times¶

In [37]:

```
AIC_df_times_ratios_dict = {}
d_df_times_ratios_dict = {}
numtolookat = 60
print('bw_method ', list(bw_scan.keys())[numtolookat])
df_times_ratios_dict = bw_scan[list(bw_scan.keys())[numtolookat]][3]

for key in df_times_ratios_dict.keys():
    print(Back.WHITE + key + Fore.RESET)
    d = df_times_ratios_dict[key].values.max()
    print('d: ', d )
    AIC = 2*2-2*np.log(df_times_ratios_dict[key].values.max())
    print('AIC: ', AIC)
    AIC_df_times_ratios_dict[key]  = AIC
    d_df_times_ratios_dict[key] = d
```

```
bw_method  6.5
sine
d:  1006571292.5
AIC:  -37.4596312653
flat
d:  30169250.6441
AIC:  -30.4446675461
```

In [38]:

```
np.log(-10)
```

```
/usr/lib/python3.6/site-packages/ipykernel/__main__.py:1: RuntimeWarning: invalid value encountered in log
  if __name__ == '__main__':
```

Out[38]:

```
nan
```

In [39]:

```
# Plotting df_times_ratios
def plot_df_times_ratios(df, fig = fig, subplot = (111), vmin = 0, vmax = 27790, key = ' '):
    ax     = fig.add_subplot(subplot);
    pcolor = ax.pcolor(df, cmap=plt.get_cmap('jet'));
    pcolor.set_clim(vmin=vmin, vmax=vmax)
    fig.colorbar(pcolor);
    plt.xticks(np.arange(0.5, len(df.columns), 1),   np.round(df.columns,2));
    plt.yticks(np.arange(0.5, len(df.index), 1), np.round(df.index,2));
    
    for label in ax.xaxis.get_ticklabels()[1::2]:
        label.set_visible(False)
    
    plt.xlabel('c');
    plt.ylabel('q');
    #plt.title(key + ': ' + 'Likelihood: empircal data coming from times-ratios for param set');
    plt.title(key);


#print(bw_scan.keys())
#df = df_times_ratios_dict.keys()
#bw_scan[bw_method] = [Ds, AICs, P, df_times_ratios_dict]
bw_method = list(bw_scan.keys())[60];     print('bw_method is: ', bw_method)
df = bw_scan[bw_method][3]
fig = plt.figure(figsize=[9,2]); iter = 0
for key in df.keys():
    df2 = deepcopy(df[key] +10*(df[key] == -10))
    df2 = df2.apply(pd.to_numeric)
    df3 = -2*np.log(df2)
    minlog  =df3.stack().nsmallest(1)
    q = list(minlog.index.values)[0][0]
    c = list(minlog.index.values)[0][1]
    vmax = np.min(-2*np.log(bw_scan[bw_method][0])); #print(vmax)
    plot_df_times_ratios(bw_scan[bw_method][4][key], fig = fig, subplot = (121+iter),vmin = -42, vmax = 100, key = key,)
    iter += 1
    # Find min log likelihood
    print(minlog)
    
fig.show()
```

```
bw_method is:  6.5
```

```
/usr/lib/python3.6/site-packages/ipykernel/__main__.py:28: RuntimeWarning: divide by zero encountered in log
```

```
0.368421  0.147368   -41.459631
dtype: float64
0.789474  0.147368   -34.444668
dtype: float64
```

In [40]:

```
-2*np.log(bw_scan[0.5][0])
```

Out[40]:

```
array([ 1338.51965104,  1130.8087916 ])
```

In [ ]:

```

```

### Purple ratio¶

In [41]:

```
AIC_df_g3_p_ratios_dict = {}
d_df_g3_p_ratios_dict = {}
for key in df_g3_p_ratios_dict.keys():
    print(Back.WHITE + key + Fore.RESET)
    d = df_g3_p_ratios_dict[key].values.max()
    print('d: ', d )
    AIC = 2*2-2*np.log(df_g3_p_ratios_dict[key].values.max())
    print('AIC: ', AIC)
    AIC_df_g3_p_ratios_dict[key]  = AIC
    d_df_g3_p_ratios_dict[key] = d
```

```
sine
d:  42.1689958674
AIC:  -3.48337051187
flat
d:  41.9719112376
AIC:  -3.47400122899
```

In [42]:

```
# Plotting df_times_ratios
def plot_df_g3_p_ratios(df_g3_p_ratios, figwidth = 4,figheight = 4, vmax = 44, key = ''):
    df_g3_p_ratios = df_g3_p_ratios.apply(pd.to_numeric)
    fig    = plt.figure(figsize=[figwidth,figheight]);
    ax     = fig.add_subplot(111);
    pcolor = ax.pcolor(df_g3_p_ratios, cmap=plt.get_cmap('jet'));
    pcolor.set_clim(vmin=0, vmax=vmax)
    fig.colorbar(pcolor);
    plt.xticks(np.arange(0.5, len(df_g3_p_ratios.columns), 1),   np.round(df_g3_p_ratios.columns,2));
    plt.yticks(np.arange(0.5, len(df_g3_p_ratios.index), 1), np.round(df_g3_p_ratios.index,2));
    plt.xlabel('c');
    plt.ylabel('q');
    plt.title(key + ': ' + 'Likelihood: empircal data coming from G3 P ratio for param set');
    fig.show()
    
# for key in df_g3_p_ratios_dict.keys():
#     plot_df_g3_p_ratios(df_g3_p_ratios_dict[key], vmax = 44,figwidth = 9, key = key)
```

### purple and times¶

In [43]:

```
# Combine the two meassure and remove 0 probability places
def likelihood_purple_times(df_g3_p_ratios, df_times_ratios):
    df_combined = df_g3_p_ratios * df_times_ratios + 0.000001 * ((df_times_ratios != -10) & (df_g3_p_ratios != -10) != 1)
    d = df_combined.values.max()
    print('d: ', d )
    AIC = 2*2-2*np.log(df_combined.values.max())
    print('AIC: ', AIC)
    return df_combined, AIC, d


df_combined_dict = {}
AIC_df_combined_dict = {}
d_df_combined_dict = {}
for key in df_g3_p_ratios_dict.keys():
    print(Back.WHITE + key + Fore.RESET)
    df_combined_dict[key], AIC_df_combined_dict[key], d_df_combined_dict[key]   = deepcopy(likelihood_purple_times(df_g3_p_ratios_dict[key], df_times_ratios_dict[key]))
```

```
sine
d:  23764443726.8
AIC:  -43.782912679
flat
d:  1034847233.16
AIC:  -37.5150393039
```

In [44]:

```
np.exp((-43.78+37.5)/2)
```

Out[44]:

```
0.043282797901965875
```

In [45]:

```
# Plotting
print(-2*np.log(2.47493296709e-06))
def plot_likelihood_purple_times(df_combined,vmin = 0, vmax = 600, figwidth = 10,figheight = 10, subplot = 111, fig = fig):
    df_combined = df_combined.apply(pd.to_numeric)
    ax     = fig.add_subplot(subplot);
    pcolor = ax.pcolor(-2*np.log(df_combined), cmap=plt.get_cmap('jet'));
    pcolor.set_clim(vmin=vmin, vmax=vmax)
    #fig.colorbar(pcolor);
    plt.xticks(np.arange(0.5, len(df_combined.columns), 1),   np.round(df_combined.columns,2));
    plt.yticks(np.arange(0.5, len(df_combined.index), 1), np.round(df_combined.index,2));
    plt.xlabel('c');
    plt.ylabel('q');
    for label in ax.xaxis.get_ticklabels()[:]:
        label.set_visible(False)
    for label in ax.xaxis.get_ticklabels()[::3]:
        label.set_visible(True)
    #plt.title('-2log(L) of empircal data coming from param set - purple and time');
    #fig.savefig('1xc0to04_sine_withtimes_samecycle.png', transparent=False)
    fig.show()
    

# fig    = plt.figure(figsize=[figwidth,figheight]); iter = 0
# for key in df_g3_p_ratios_dict.keys():
#     plot_likelihood_purple_times(df_combined_dict[key], vmin = -43, vmax = 100, subplot = 121+iter, fig = fig); iter +=1
#     #fig = plt.gcf()
#     #fig.savefig('paramscan'+str(key)+'.svg')
# fig.show()
    
figwidth = 5
figheight = 6
fig    = plt.figure(figsize=[figwidth,figheight])
plot_likelihood_purple_times(df_combined_dict['sine'], vmin = -50, vmax = 100, subplot = 111, fig = fig); iter +=1
#fig.savefig('z_paramscan_sine_nobar.svg')
fig.show()
    

fig    = plt.figure(figsize=[figwidth,figheight])
plot_likelihood_purple_times(df_combined_dict['flat'], vmin = -50, vmax = 100, subplot = 111, fig = fig); iter +=1
#fig.savefig('z_paramscan_flat_nobar.svg')
fig.show()
```

```
25.8185944926
```

```
/usr/lib/python3.6/site-packages/ipykernel/__main__.py:6: RuntimeWarning: divide by zero encountered in log
```

In [46]:

```
df = deepcopy(df_combined_dict['sine'].apply(pd.to_numeric))
np.min(np.min(-2*np.log(df)))
```

```
/usr/lib/python3.6/site-packages/ipykernel/__main__.py:2: RuntimeWarning: divide by zero encountered in log
  from ipykernel import kernelapp as app
```

Out[46]:

```
-47.782912678984871
```

### purple, times, blue¶

In [47]:

```
# Combine the two meassure and remove 0 probability places 
def likelihood_purple_blue_times(df_g3_p_ratios, df_times_ratios, df_g2_b_ratios):
    df_combined2 = df_g3_p_ratios * df_times_ratios * df_g2_b_ratios -10 * ((df_times_ratios != -10) & (df_g3_p_ratios != -10) & (df_g2_b_ratios != -10) != 1)
    d = df_combined2.values.max()
    print('d: ', d )
    AIC = 2*2-2*np.log(df_combined2.values.max() )
    print('AIC: ', AIC)
    return df_combined2, AIC, d

# Plotting
def plot_likelihood_purple_blue_times(df_combined2,vmin = -100, vmax = 600, figwidth = 4,figheight = 4):
    fig    = plt.figure(figsize=[figwidth+3,figheight+3]);
    ax     = fig.add_subplot(111);
    pcolor = ax.pcolor(-2*np.log(df_combined2.apply(pd.to_numeric)), cmap=plt.get_cmap('jet'));
    pcolor.set_clim(vmin=vmin, vmax=vmax)
    fig.colorbar(pcolor);
    plt.xticks(np.arange(0.5, len(df_combined2.columns), 1),   np.round(df_combined2.columns,2));
    plt.yticks(np.arange(0.5, len(df_combined2.index), 1), np.round(df_combined2.index,2));
    plt.xlabel('c');
    plt.ylabel('q');
    plt.title('Relative probab of empircal data coming from param set - sine - same cellcycle for P, G and B - all 3');
    fig.savefig('1xc0to04_sine_withtimes_samecycle.png', transparent=False)
    fig.show()
    max(df_combined2.max())
```

In [ ]:

```

```

In [48]:

```
df_combined_dict2 = {}
AIC_df_combined2_dict = {}
d_df_combined2_dict = {}
for key in df_g3_p_ratios_dict.keys():
    print(Back.WHITE + key + Fore.RESET)
    df_combined_dict2[key], AIC_df_combined2_dict[key], d_df_combined2_dict[key] = deepcopy(likelihood_purple_blue_times(df_g3_p_ratios_dict[key], df_times_ratios_dict[key], df_g2_b_ratios_dict[key]))
    
plot_likelihood_purple_blue_times(df_combined_dict2['sine'],vmin = -50,  vmax = 100, figwidth = 4,figheight = 4)  
plot_likelihood_purple_blue_times(df_combined_dict2['flat'],vmin = -50,  vmax = 100, figwidth = 4,figheight = 4)
```

```
sine
d:  1.55171521094e+12
AIC:  -52.1407640455
flat
d:  72154924765.6
AIC:  -46.0041627543
```

```
/usr/lib/python3.6/site-packages/ipykernel/__main__.py:14: RuntimeWarning: divide by zero encountered in log
```

```
/usr/lib/python3.6/site-packages/ipykernel/__main__.py:14: RuntimeWarning: invalid value encountered in log
/usr/lib/python3.6/site-packages/matplotlib/colors.py:494: RuntimeWarning: invalid value encountered in less
  cbook._putmask(xa, xa < 0.0, -1)
```

# AIC¶

In [49]:

```
df_AIC_df_times_ratios = pd.DataFrame(index=list(AIC_df_times_ratios_dict.keys()), columns=list(AIC_df_times_ratios_dict.keys()))
df_AIC_df_times_ratios.columns.name = '# time only #'
for key_i in AIC_df_times_ratios_dict.keys():
    for key_j in AIC_df_times_ratios_dict.keys():
        compare = np.exp((AIC_df_times_ratios_dict[key_i] - AIC_df_times_ratios_dict[key_j])/(2))
        df_AIC_df_times_ratios.loc[key_i,key_j] = compare
df_AIC_df_times_ratios
```

Out[49]:

| # time only # | sine | flat |
| --- | --- | --- |
| sine | 1 | 0.0299723 |
| flat | 33.3641 | 1 |

In [50]:

```
df_AIC_df_g3_p_ratios = pd.DataFrame(index=list(AIC_df_g3_p_ratios_dict.keys()), columns=list(AIC_df_g3_p_ratios_dict.keys()))
df_AIC_df_g3_p_ratios.columns.name = '# purple only #'
for key_i in AIC_df_g3_p_ratios_dict.keys():
    for key_j in AIC_df_g3_p_ratios_dict.keys():
        compare = np.exp((AIC_df_g3_p_ratios_dict[key_i] - AIC_df_g3_p_ratios_dict[key_j])/(2))
        df_AIC_df_g3_p_ratios.loc[key_i,key_j] = compare
df_AIC_df_g3_p_ratios
```

Out[50]:

| # purple only # | sine | flat |
| --- | --- | --- |
| sine | 1 | 0.995326 |
| flat | 1.0047 | 1 |

In [51]:

```
df_AIC_df_combined = pd.DataFrame(index=list(AIC_df_combined_dict.keys()), columns=list(AIC_df_combined_dict.keys()))
df_AIC_df_combined.columns.name = '# purple and time #'
for key_i in AIC_df_combined_dict.keys():
    for key_j in AIC_df_combined_dict.keys():
        compare = np.exp((AIC_df_combined_dict[key_i] - AIC_df_combined_dict[key_j])/(2))
        df_AIC_df_combined.loc[key_i,key_j] = compare
df_AIC_df_combined
```

Out[51]:

| # purple and time # | sine | flat |
| --- | --- | --- |
| sine | 1 | 0.043546 |
| flat | 22.9642 | 1 |

In [52]:

```
df_AIC_df_combined2 = pd.DataFrame(index=list(AIC_df_combined2_dict.keys()), columns=list(AIC_df_combined2_dict.keys()))
df_AIC_df_combined2.columns.name = '# purple, blue and time #'
for key_i in AIC_df_combined2_dict.keys():
    for key_j in AIC_df_combined2_dict.keys():
        compare = np.exp((AIC_df_combined2_dict[key_i] - AIC_df_combined2_dict[key_j])/(2))
        df_AIC_df_combined2.loc[key_i,key_j] = compare     
df_AIC_df_combined2
```

Out[52]:

| # purple, blue and time # | sine | flat |
| --- | --- | --- |
| sine | 1 | 0.0465001 |
| flat | 21.5053 | 1 |

In [ ]:

```

```

In [ ]:

```

```

In [ ]:

```

```

# Show 2D kernels¶

In [53]:

```
# Show 2D kernels
def show_2d_kernels(paramscan_times_df2, q_n = 5, c_n = 5, bw_method = 0.5, figwidth = 3,figheight = 3):
    #q_n = 6; c_n = 6
    q_i = q_index[q_n]
    c_i = c_index[c_n]
    b_ratios_at_timepoint, g_ratios_at_timepoint, p_ratios_at_timepoint, B_freq, G_freq, P_freq = ratios_at_timepoints2(q_i, c_i, paramscan_times_df2)

    fig = plt.figure(figsize=[figwidth,figheight])
    # for Figure
    for timepoint_n in range(4):
        b = b_ratios_at_timepoint[timepoint_n]
        p = p_ratios_at_timepoint[timepoint_n]
        values = np.vstack([b, p])
        kernel = stats.gaussian_kde(values, bw_method= bw_method)

        xmin = b.min()
        xmax = b.max()
        ymin = p.min()
        ymax = p.max()
        X, Y = np.mgrid[xmin:xmax:100j, ymin:ymax:100j]
        positions = np.vstack([X.ravel(), Y.ravel()])
        Z = np.reshape(kernel(positions).T, X.shape)

        # Figure
        ax = fig.add_subplot(2,4,5+timepoint_n)
        ax.imshow(np.rot90(Z), cmap=plt.cm.gist_earth_r,
                  extent=[xmin, xmax, ymin, ymax])
        ax.plot(b, p, 'k.', markersize=2)
        ax.set_xlim([xmin, xmax])
        ax.set_ylim([ymin, ymax])
        ax.set_xlabel('B freq')
        ax.set_ylabel('P freq')
        ax.set_aspect('auto')

    ax2 = fig.add_subplot(211)
    x = [1,2,3,4]
    ax2.bar(x, B_freq,                      color='#00BFFF',lw=0, label = 'B')
    ax2.bar(x, G_freq, bottom=B_freq,       color='#00FF00',lw=0, label = 'G')
    ax2.bar(x, P_freq, bottom=B_freq+G_freq,color='#800080',lw=0, label = 'P')
    plt.xticks(np.array(x)+0.4, [10.5,11.5,12.5,14.5], rotation='horizontal')
    ax2.set_title('q: '+str(q_i)+' & c: '+str(c_i))

    fig.show()
```

In [54]:

```
q_n = 4; c_n = 4
q_i = q_index[q_n]
c_i = c_index[c_n]
b_ratios_at_timepoint, g_ratios_at_timepoint, p_ratios_at_timepoint, B_freq, G_freq, P_freq = ratios_at_timepoints2(q_i, c_i, paramscan_times_df2_dict['sine'])
i = random.sample(range(len(b_ratios_at_timepoint[0][:])),1)
B_freq = np.array([b_ratios_at_timepoint[j][i][0] for j in range(4)])
G_freq = np.array([g_ratios_at_timepoint[j][i][0] for j in range(4)])
P_freq = np.array([p_ratios_at_timepoint[j][i][0] for j in range(4)])
print(B_freq)
print(G_freq)
print(P_freq)

fig = plt.figure()
ax2 = fig.add_subplot(111)
x = [1,2,3,4]
ax2.bar(x, B_freq,                      color='#00BFFF',lw=0, label = 'B')
ax2.bar(x, G_freq, bottom=B_freq,       color='#00FF00',lw=0, label = 'G')
ax2.bar(x, P_freq, bottom=B_freq+G_freq,color='#800080',lw=0, label = 'P')
plt.xticks(np.array(x)-0.0, ['E10.5','E11.5','E12.5','E14.5'], rotation='horizontal')
plt.ylabel('Frequency')
#ax2.set_title('q: '+str(q_i)+' & c: '+str(c_i))
fig.show()
```

```
[ 0.2         0.16666667  0.14705882  0.15286624]
[ 0.8         0.83333333  0.82352941  0.77070064]
[ 0.          0.          0.02941176  0.07643312]
```

In [55]:

```
#fig = plt.gcf()
#fig.savefig('timesplot_44_label_6.svg')
```

In [56]:

```
bw_method = 0.5
show_2d_kernels(paramscan_times_df2_dict['sine']   , q_n = 4, c_n = 4, bw_method = bw_method, figwidth = 6,figheight = 3)
#fig = plt.gcf()
#fig.savefig('z_2dKernels05.svg')
#show_2d_kernels(paramscan_times_df2_dict['sine_notfixed'], q_n = 4, c_n = 4, bw_method = bw_method, figwidth = 6,figheight = 3)


#show_2d_kernels(paramscan_times_df2_dict['flat']         , q_n = 4, c_n = 4, bw_method = bw_method, figwidth = 6,figheight = 3)
```

In [99]:

```
iter = 0
fig = plt.figure(figsize=[figwidth,figheight])
keys = paramscan_times_df2_dict.keys()
keys = ['sine']
for key in keys:

    q_n = {'sine':7, 'flat':16}
    c_n = {'sine':6, 'flat':6}
    q_i = q_index[q_n[key]]
    c_i = c_index[c_n[key]]
    
    times_df = paramscan_times_df2_dict[key]
    kernel_105, kernel_115, kernel_125, kernel_145, b_ratios_in_all_minipancreas_np, p_ratios_in_all_minipancreas_np, g_ratios_in_all_minipancreas_np = get_2dkernels_for_params_minipancreas_with_ratios(q_i, c_i, times_df, 
     bw_method = 10.50, bundle_size = 100, bundle_type = 'conservative', n_bootstraps_to_create = 1000) 

    kernels = [kernel_105, kernel_115, kernel_125, kernel_145]

    # for Figure

    p_105_0 = [np.array(figS7_ratios.loc['10.5','B/T'])[0], np.array(figS7_ratios.loc['10.5','P/T'])[0]]
    p_115_0 = [np.array(figS7_ratios.loc['11.5','B/T'])[0], np.array(figS7_ratios.loc['11.5','P/T'])[0]]
    p_125_0 = [np.array(figS7_ratios.loc['12.5','B/T'])[0], np.array(figS7_ratios.loc['12.5','P/T'])[0]]
    p_145_0 = [np.array(figS7_ratios.loc['14.5','B/T'])[0], np.array(figS7_ratios.loc['14.5','P/T'])[0]]
    p_0 = [p_105_0, p_115_0, p_125_0, p_145_0]

    p_105_1 = [np.array(figS7_ratios.loc['10.5','B/T'])[1], np.array(figS7_ratios.loc['10.5','P/T'])[1]]
    p_115_1 = [np.array(figS7_ratios.loc['11.5','B/T'])[1], np.array(figS7_ratios.loc['11.5','P/T'])[1]]
    p_125_1 = [np.array(figS7_ratios.loc['12.5','B/T'])[1], np.array(figS7_ratios.loc['12.5','P/T'])[1]]
    p_145_1 = [np.array(figS7_ratios.loc['14.5','B/T'])[1], np.array(figS7_ratios.loc['14.5','P/T'])[1]]
    p_1 = [p_105_1, p_115_1, p_125_1, p_145_1]

    
    for timepoint_n in range(4):
        b = b_ratios_in_all_minipancreas_np[:,timepoint_n]
        p = p_ratios_in_all_minipancreas_np[:,timepoint_n]
        values = np.vstack([b, p])
        kernel = kernels[timepoint_n]

        xmin = 0
        xmax = 1
        ymin = 0
        ymax = 1
    #     xmin = b.min()
    #     xmax = b.max()
    #     ymin = p.min()
    #     ymax = p.max()
        X, Y = np.mgrid[xmin:xmax:100j, ymin:ymax:100j]
        positions = np.vstack([X.ravel(), Y.ravel()])
        Z = np.reshape(kernel(positions).T, X.shape)

        # Figure
        ax = fig.add_subplot(len(keys),4,(iter*4)+1+timepoint_n)
        cax = ax.imshow(np.rot90(Z), cmap=plt.cm.gist_earth_r,
                  extent=[xmin, xmax, ymin, ymax])
        #ax.plot(b, p, 'w.', markersize=2)
        #ax.plot(p_0[timepoint_n][0],p_0[timepoint_n][1], 'ro', markersize=2)
        #ax.plot(p_1[timepoint_n][0],p_1[timepoint_n][1], 'ro', markersize=2)
        ax.set_xlim([xmin, xmax])
        ax.set_ylim([ymin, ymax])
        
        #if timepoint_n == 0:
            #ax.set_ylabel('P freq')
            #ax.set_xlabel('B freq')
        ax.set_aspect('auto')
        #ax.set_title(key)
        #if timepoint_n == 3:
        fig.colorbar(cax, orientation='horizontal')        
    iter += 1
fig.show()
```

In [100]:

```
fig.savefig('z_2Dkde_2.svg')
```

In [60]:

```
print( np.std( b_ratios_in_all_minipancreas_np[:,timepoint_n] ) )
print( np.std( p_ratios_in_all_minipancreas_np[:,timepoint_n] ) )
```

```
0.00551456548859
0.0169956260674
```

In [61]:

```
print( max(bw_scan[bw_method][3]['flat'].idxmax(axis = 0)) )
print( max(bw_scan[bw_method][3]['flat'].idxmax(axis = 1)) )
```

```
0.736842105263
0.189473684211
```

### Test neighbor dist¶

In [62]:

```
# q_n = 4; c_n = 4
# q_i = q_index[q_n]
# c_i = c_index[c_n]
# b_ratios_at_timepoint, g_ratios_at_timepoint, p_ratios_at_timepoint, B_freq, G_freq, P_freq = ratios_at_timepoints(q_i, c_i, paramscan_times_df2_dict['sine'])

# min_dists_at_time = []
# dists_no_zero_at_time = [[],[],[],[]]
# for timepoint_n in range(4):
#     b = b_ratios_at_timepoint[timepoint_n]
#     p = p_ratios_at_timepoint[timepoint_n]
#     min_dists = []
#     dists_no_zero = []
#     for i in range(len(b)):
#         dists = []
#         for j in range(len(b)):
#             b_self = b[i]
#             p_self = p[i]
#             b_other = b[j]
#             p_other = p[j]
#             dists.append(np.sqrt((b_self-b_other)**2 + (p_self-p_other)**2))
#         dists_not_zero = np.array(dists)[np.array(dists) != 0]
#         min_dists.append(dists_not_zero.min())
#         dists_no_zero_at_time[timepoint_n] = dists_no_zero_at_time[timepoint_n] + list(dists_not_zero)
#     min_dists_at_time.append(min_dists)
```

In [63]:

```
# bins = 20
# fig = plt.figure(figsize=[14,4])
# ax1 = fig.add_subplot(1,4,1)
# ax1.hist(min_dists_at_time[0], bins = bins, normed=1, color = 'gray')
# ax1.set_title('10.5')
# ax1.set_xlabel('distance in ratio plane')
# ax1.set_ylabel('frequency')
# ax2 = fig.add_subplot(1,4,2)
# ax2.hist(min_dists_at_time[1], bins = bins, normed=1, color = 'gray')
# ax2.set_title('11.5')
# ax2.set_xlabel('distance in ratio plane')
# ax3 = fig.add_subplot(1,4,3)
# ax3.hist(min_dists_at_time[2], bins = bins, normed=1, color = 'gray')
# ax3.set_title('12.5')
# ax3.set_xlabel('distance in ratio plane')
# ax4 = fig.add_subplot(1,4,4)
# ax4.hist(min_dists_at_time[3], bins = bins, normed=1, color = 'gray')
# ax4.set_title('14.5')
# ax4.set_xlabel('distance in ratio plane')
# fig.suptitle("Distance to closest neighbour in PTF1A-NEUROG3 ratio space", fontsize=12)
# fig.show()
# fig.savefig('ratioplane_closest.svg')

# bins = 20
# fig = plt.figure(figsize=[14,4])
# ax1 = fig.add_subplot(1,4,1)
# ax1.hist(dists_no_zero_at_time[0], bins = bins, normed=1, color = 'gray')
# ax1.set_title('10.5')
# ax1.set_xlabel('distance in ratio plane')
# ax1.set_ylabel('frequency')
# ax2 = fig.add_subplot(1,4,2)
# ax2.hist(dists_no_zero_at_time[1], bins = bins, normed=1, color = 'gray')
# ax2.set_title('11.5')
# ax2.set_xlabel('distance in ratio plane')
# ax3 = fig.add_subplot(1,4,3)
# ax3.hist(dists_no_zero_at_time[3], bins = bins, normed=1, color = 'gray')
# ax3.set_title('12.5')
# ax3.set_xlabel('distance in ratio plane')
# ax4 = fig.add_subplot(1,4,4)
# ax4.hist(dists_no_zero_at_time[3], bins = bins, normed=1, color = 'gray')
# ax4.set_title('14.5')
# ax4.set_xlabel('distance in ratio plane')
# fig.suptitle("Distance of all cells to all other in PTF1A-NEUROG3 ratio space", fontsize=12)
# fig.show()
# fig.savefig('ratioplane_alltoall.svg')
```

In [ ]:

```

```

In [ ]:

```

```

In [ ]:

```

```

In [ ]:

```

```

In [ ]:

```

```

In [ ]:

```

```

In [ ]:

```

```

In [ ]:

```

```
